# Early life bifidobacterial mother–infant transmission: greater contribution from the infant gut to human milk revealed by microbiomic and culture-based methods

**DOI:** 10.1128/msystems.00480-25

**Published:** 2025-06-25

**Authors:** Simou Wu, Gang Luo, Fengling Jiang, Wen Jia, Jinxing Li, Ting Huang, Xuguang Zhang, Yuejian Mao, Shengpeng Su, Weiwei Han, Fang He, Ruyue Cheng

**Affiliations:** 1 Department of Nutrition and Food Hygiene, West China School of Public Health and West China Fourth Hospital, Sichuan University, Chengdu, Sichuan, China; 2 Sichuan Tianfu New Area Public Health Center, Chengdu, Sichuan, China; 3 Global R&D Innovation Center, Inner Mongolia Mengniu Dairy (Group) Co. Ltd., Hohhot, Inner Mongolia, China; Northern Arizona University, Flagstaff, Arizona, USA

**Keywords:** early life, mother–infant transmission, human milk microbiota, infant gut microbiota, *Bifidobacterium*

## Abstract

The colonization and development of the gut microbiota during early life, especially *Bifidobacterium*, may be influenced by maternal bacterial communities, including those of human milk. However, the interaction of bacteria in mother–infant dyads during breastfeeding remains unclear. This study focused primarily on the characteristics and dynamics of the infant gut and human milk microbiota within the first month of life on the basis of a birth cohort and explored the interaction of the microbiota derived from the two niches by sequencing and culture-based methods, especially *Bifidobacterium,* as the representative dominator in the infant gut. Infant feces and human milk samples from 21 mother–infant dyads were collected on days 0, 7, and 30 postpartum. The bacterial composition was identified by sequencing the 16S rRNA gene, and the contributions of the bacterial communities were estimated via SourceTracker2. Bifidobacterial strains were isolated from infant feces and human milk via culture-based methods. The suspected strains were identified through Sanger sequencing and genotyped via multilocus sequence typing (MLST). The bacterial communities were distinct between infant feces and human milk. Human milk microbes contribute 63.89%–77.61% to the infant’s gut within the first month of life, whereas *Bifidobacterium* in the infant’s gut contributes more (80.18%–84.30%) to human milk. A total of 60 bifidobacterial isolates were obtained from 10 pairs of mother–infant samples, 48 isolates from 10 out of 27 infant feces samples, and 12 isolates from 4 out of 27 human milk samples. Among these, 30 isolates were identified as *Bifidobacterium breve,* and 18 were identified as *B. longum* subsp. *longum*. Strains belonging to *B. breve* from a single mother–infant pair were found to be monophyletic (ST: BRE-1), whereas this strain was found much earlier in infant feces across the three time points (collected on days 0, 7, and 30) than in human milk (collected on day 30). Our data suggest that during very early breastfeeding, human milk contributes a significant proportion of the overall bacterial population to the infant’s gut, whereas the infant’s gut selectively contributes a greater proportion of *Bifidobacterium* to human milk. Certain bifidobacterial strains, such as *B. breve*, are retrogradely transmitted from the infant’s gut to the mother’s human milk during breastfeeding, implying a potential challenge regarding the reliability of the source when potential probiotics are isolated from human milk.

**IMPORTANCE:**

Understanding how microbes, especially beneficial bacteria such as *Bifidobacterium,* are shared between mothers and infants during breastfeeding is crucial for promoting infant health. Although most research has focused on transmission from mother to child, our study reveals a novel and significant reverse route: from the infant gut to breast milk. By combining microbiome sequencing with culture-based techniques, we provide evidence that specific strains of *Bifidobacterium*, especially *B. breve*, may transmit back to the mother during breastfeeding. This insight reshapes our understanding of microbial exchange within the mother–infant dyad and highlights breastfeeding as a bidirectional process that influences both maternal and infant microbiota. These findings may have important implications for designing probiotics and supporting early-life microbial development through maternal health interventions.

## INTRODUCTION

Early life represents a critical period for the establishment of the gut microbiota. During childbirth, a substantial number of microbes begin to colonize the neonatal gut, with the gut microbiota generally stabilizing before the age of 1 year. Delayed colonization or dysbiosis in early life is closely linked to diseases such as allergies and obesity (1, 2). The transmission of the gut microbiota from the mother to the infant plays a pivotal role in the acquisition, development, and potential intervention of the infant’s gut microbiota. This process is influenced by various factors, including maternal age, mode of delivery, feeding practices, and environmental exposure (3). Emerging evidence suggests that microbial colonization may begin prenatally. Walker et al. (4) reviewed multiple studies supporting the hypothesis of *in utero* transmission, proposing that bacteria from the maternal gut, oral cavity, or vaginal microbiota could translocate to the fetal environment via the bloodstream or amniotic fluid ingestion. Our previous *in vivo* study demonstrated that the maternal gut and placenta harbor distinct microbiota compositions, which partially influence the immune responses of both mothers and their offspring (5). Although vaginal strains colonize the infant gut only transiently, maternal gut-derived strains proved more persistent in the infant gut (6, 7). Postnatally, the impact of breastfeeding on shaping the infant’s gut microbiota has garnered increasing attention (8). However, research on the influence of human milk on infant gut microbiota remains relatively underexplored. Understanding the dynamics and mechanisms of microbial transfer through human milk is crucial for comprehending its role in early life health.

Human milk is not only a source of nutrients for infants but also provides beneficial bacteria that help develop the infant’s gut microbiota. Studies have shown that although the maternal gut microbiome is the primary contributor to infant gut colonization, the breast milk microbiota also plays a significant role in shaping the infant gut microbiome (7, 9–11). However, even in naturally delivered, exclusively breastfed infants, differences remain between the gut bacteria in infants and those in breast milk (12). For example, *Bifidobacterium* dominates the infant’s gut within the first 6 months, whereas *Staphylococcus* and *Streptococcus* are more common in human milk (13). Moreover, the composition of human milk bacteria also changes over time, with different bacteria present in colostrum, transitional milk, and mature milk (14). This changeable developmental pattern of the human milk microbiota may be synchronous with the more dynamic gut microbiota of infants over time. This finding suggests that although these two niches are distinct, the interaction between the infant’s gut and the human milk microbiota continues to occur. Currently, most research has focused on how breastfeeding affects the infant’s gut microbiota, with less attention given to the interaction between the two niches during breastfeeding.

*Bifidobacterium* is the dominant taxon in the intestines of breastfed infants. As an important source of probiotics, *Bifidobacterium* species play crucial physiological roles in the maturation of the immune system, the regulation of the gut microbiota, and nutrient metabolism (15). Infant-type bifidobacteria can specifically utilize indigestible human milk oligosaccharides (HMOs) to produce short-chain fatty acids (SCFAs), which, through a process known as “cross-feeding,” help in the formation of the gut microbiota, maintain gut environment stability, and inhibit pathogenic bacterial invasion, thus exerting probiotic effects in the gut (16). An increasing number of studies have focused on bifidobacterial strains derived from human milk, and some studies have isolated strains from human milk via culture methods (17). Additionally, studies that isolated bifidobacteria from both human milk and infant feces found that certain strains were present in both sources (18, 19), indicating the potential role of human milk in the colonization of certain bifidobacteria in the infant gut. However, we still know very little about the timing of the appearance of bifidobacteria in human milk and whether human milk is the initial source of colonization. Some researchers have proposed the “enteromammary” axis hypothesis, suggesting that during late pregnancy and lactation, dendritic cells penetrate the gut epithelium, capture bifidobacteria from the mother’s gut, and deliver it to the mammary glands through macrophage presentation (20). Other studies suggest that during breastfeeding, bifidobacterial strains in the infant’s gut may retrograde into the mammary glands with the backflow of milk (21). Future research is needed to explore the potential transmission pathway of bifidobacteria between human milk and the infant gut.

This study focuses primarily on the characteristics and dynamics of the infant gut and human milk microbiota within the first month of life in a mother–infant cohort from southwest China. This study explored the potential bidirectional influence between the breast milk microbiota and the infant gut microbiota, suggesting possible interactions between these two niches. Additionally, this study explored the composition and potential transmission pathways of bifidobacteria derived from both human milk and the infant’s gut via both microbiome-bioinformatic and culture-based methods. These findings provide new insights into the microbial transmission between the infant gut and mother’s human milk during breastfeeding, as well as the isolation source of bifidobacterial strains.

## MATERIALS AND METHODS

### Subjects

This study was conducted as a birth cohort investigation, where mother–infant pairs were enlisted from November 2020 to July 2021 at the West China Second Hospital of Sichuan University, Chengdu, China. We collected information on the participants both before delivery and 1 month after delivery using questionnaires. This information included basic demographic details, feeding patterns, medication, and hospitalization records of both mothers and infants. All participants were recruited after signing informed consent.

The eligibility criteria for participation included the following: (i) a prepregnancy body mass index (BMI) within the normal range (18.5–23.9 kg/m^2^); (ii) absence of *in vitro* fertilization (IVF) intervention; (iii) no history of diabetes, hypertension, or infectious, autoimmune, or genetic disorders in the mother before pregnancy; and (d) delivery of a full-term infant without congenital or hereditary conditions. The exclusion criteria were as follows: (i) delivery by Cesarean section; (ii) maternal intake of antibiotics or pro-/pre-biotics supplements within 1 month preceding labor; (iii) diagnosis of severe illnesses in infants during the follow-up; and (iv) nonexclusive breastfeeding of the infant throughout the follow-up period.

### Sample collection and processing

Sterile sampling tubes were used to collect infant feces and human milk samples on days 0, 7, and 30 after newborn birth. The sampling process was divided into in-hospital and post-discharge collections. During the hospital stay, trained investigators used sterile tubes for sampling and provided training for each pregnant woman. For post-discharge periods, mothers were reminded to self-sample the day before the corresponding sampling time point. The samples were kept at 4°C in ice bags and transported to the laboratory within 2 h. Upon arrival, the sample collection time, weight, and identification number were recorded. The samples were stored at −80°C for preservation.

### Fecal and human milk bacterial DNA extraction

Fecal bacterial DNA was extracted using the Fecal DNA Extraction Kit (DP328-02, TIANGEN, Beijing, China), with an additional bead-beating step to enhance bacterial cell lysis and improve DNA yield. Human milk bacterial DNA was extracted using the Magnetic Soil and Stool DNA Kit (DP712-01, TIANGEN, Beijing, China), with modifications including an increased initial sample volume and extended lysis incubation time to optimize DNA recovery from the low-biomass milk samples. All procedures were conducted in reference to the manufacturer’s manual with these slight modifications. The concentration and purity of the purified DNA were subsequently assessed using a UV–Vis microvolume spectrophotometer (NanoDrop 2000, Thermo Fisher Scientific, Inc., USA). The purified DNA samples were then preserved at −80°C for later use.

### 16S rRNA sequencing and bioinformatic analysis

The 5′ ends of the primers were tagged to each sample with specific barcodes, and the V3–V4 region of the 16S rRNA gene was amplified with the primers 338F (5′-ACTCCTACGGGAGGCAGCAG-3′) and 806R (5′-GGACTACHVGGGTWTCTAAT-3′). The polymerase chain reaction (PCR) products were confirmed by 2% agarose gel electrophoresis and then purified. The resulting amplicon library was then evaluated and sequenced using an Illumina MiSeq system (Illumina Inc., CA, USA).

Raw data were obtained through base calling using the bcl2fastq software (v.1.8.4, available from https://support.illumina.com). Reads with missing, incorrect, or conflicting barcodes were trimmed using Trimmomatic (v.0.36) (22). The QIIME2-DADA2 pipeline (qiime2-2021.8) (23) was subsequently employed to filter and merge paired-end reads while removing chimeric sequences to obtain clean reads. A feature table and feature sequences were then constructed following the DADA2 denoising and inference pipeline (24). High-quality sequences were clustered into amplicon sequence variants (ASVs) using Qiime 2. The ASVs were annotated based on the SILVA database (v.138), and taxonomic assignments were considered reliable when bootstrap confidence values were above 0.75 (25). An ASV-abundance table and a phylogenetic tree were subsequently generated. Alpha diversity, including the observed ASVs and the ACE, Chao1, Shannon, and Simpson indices, was calculated via QIIME 2, and rarefaction curves of alpha diversity were generated using QIIME 2. Beta-diversity was assessed on the basis of all ASVs, employing non-metric multidimensional scaling (NMDS) with the Bray‒Curtis dissimilarity algorithm. Data visualization was conducted in R (v.4.0.4) using the Phyloseq package (v.1.20.0). On the basis of the ASV table, QIIME 2 was used to calculate the community composition and species abundance at different taxonomic levels.

### Bacterial source tracking

SourceTracker is a Bayesian approach to estimate the sources and proportions of contaminants in a given community (sink samples) that come from a potential source community (source samples) (26). We applied SourceTracker (v.0.9.1) to estimate the contribution of bacteria between human milk and infant feces at different time points. To further investigate the contribution of *Bifidobacterium* to the microbial communities in the sink samples, we extracted *Bifidobacterium*-related ASVs from the total bacterial ASVs of each sample and then conducted source tracking analysis of *Bifidobacterium*-related ASVs against the total bacterial ASVs, as well as *Bifidobacterium*-related ASVs against the *Bifidobacterium*-related ASVs between different groups.

### Isolation and identification of *Bifidobacterium*

Human milk and infant feces were serially diluted at proper times with dilution water (content in 1 L: 4.5 g KH_2_PO_4_, 6.0 g Na_2_HPO_4_, 0.5 g L-cysteine, 0.5 g Tween-80 and 1.0 g agar [Solarbio Science & Technology Co., Ltd., Beijing, China]), inoculated onto TOS propionate agar plates (EIKEN CHEMICAL Co., Ltd., Tokyo, Japan), and incubated anaerobically at 37°C for 48–72 h in an anaerobic chamber (MITSUBISHI GAS CHEMICAL Co., Inc., Tokyo, Japan). For human milk samples, the original liquid and a 10-fold serial dilution series (from 10^−^¹ to 10^−^³) were incubated, whereas for infant fecal samples, a 10-fold serial dilution series ranging from 10^−^⁷ to 10^−^⁹ was incubated. Quantification of bifidobacteria strains in samples was assessed using the flat colony counting method after incubation for 48–72 h. For each sample, 2–8 colonies showing suspected morphologies on the medium were isolated and purified for subsequent analyses.

DNA was extracted from each isolate through the boiling lysis method, with slight modifications. Briefly, each isolate was suspended in 100 µL of TE buffer (10 mM Tris·HCl, 1 mM EDTA; pH 8.0) and then boiled in a water bath at 100°C for 5 min. After cooling, the mixture was centrifuged at 12,000 rpm for 5 min, and the supernatant was collected as DNA. Isolates were identified at the species level through polymerase chain reaction (PCR) sequencing of the 16S rRNA gene by using the universal primers 338F (5′-ACTCCTACGGGAGGCAGCAG-3′) and 806R (5′-GGACTACHVGGGTWTCTAAT-3′). The PCR conditions used were 2 min at 94°C and 35 cycles of 30 s at 94°C, 30 s at 55°C, and 30 s at 72°C, followed by 2 min at 72°C. Amplification was carried out using a ProFlex 96-well PCR Thermocycler (Thermo Fisher Scientific Inc., Massachusetts, USA). The resulting sequences were used to search sequences deposited in the NCBI database via the BLAST algorithm (https://www.ncbi.nlm.nih.gov/), and the identities of the isolates were determined on the basis of the highest scores of Per. Ident value (>99%).

### Multilocus sequence typing (MLST) analysis

Multilocus sequence typing (MLST) analysis was performed to investigate the identity among the isolated strains isolated from different samples and differentiate duplicate isolates from the same sample. Seven housekeeping genes, *clpC, fusA, gyrB, purF, rpoB, ileS,* and *rplB,* were used to distinguish *Bifidobacterium* at the strain level, as proposed by Santos and Ochman and Ventura et al. (27, 28). The primer sequences are provided in Table 1. The PCR amplification program used was as follows: 5 min at 95°C; 30 cycles of 30 s at 95°C, 30 s at 60°C, and 60 s at 72°C; and 10 min at 72°C for the *clpC, fusA, gyrB, purF,* and *rplB* genes. The PCR amplification program for the genes *ileS* and *rpoB* was at annealing temperatures of 55°C (29). BioNumerics software 8.0 (Applied Maths, Sint-Martens-Latem, Belgium) was used for phylogenetic analyses. For the MLST data, the sequences obtained from the seven housekeeping genes were aligned and compared. Each unique gene sequence was assigned an allele number, and each distinct combination of these seven allele numbers was designated a sequence type (ST). Cluster analysis based on allelic profiles was then performed using the unweighted pair group method with arithmetic means (UPGMA) algorithm, implemented in BioNumerics, to analyze the categorical coefficient.

**TABLE 1:** Genes and sequencing primers for MLST analysis

Locus	Length (bp)	Primers (5′−3′)
*clpC*	600	clpC-uni: GAGTACCGCAAGTACATCGAG clpC-rev: CATCCTCATCGTCGAACAGGAAC
*fusA*	666	fusAB3: ATCGGCATCATGGCYCACATYGAT fusAB4: CCAGCATCGGCTGMACRCCCTT
*gyrB*	627	gyrBB3: AGCTGCACGCBGGCGGCAAGTTCG gyrBB4: GTTGCCGAGCTTGGTCTTGGTCTG
*purF*	591	purF-uni: CATTCGAACTCCGACACCGA purF-rev: GTGGGGTAGTCGCCGTTG
*rpoB*	501	rpoBB3: GGCGAGCTGATCCAGAACCA rpoBB4: GCATCCTCGTAGTTGTASCC
*ileS*	489	ileSB3: ATCCCGCGYTACCAGACSATG ileSB4: CGGTCGACGTAGTCGGCG
*rplB*	357	rplBB3: GGACAAGGACGGCRTSCCSGCCAA rplBB4: ACGACCRCCGTGCGGGTGRTCGAC

### Statistical analysis

GraphPad Prism (v.9.4.1) and R (v.4.0.4) were used for data processing and visualization. For quantitative variables that met the assumption of homogeneity, one-way ANOVA was used to analyze the differences between groups, and the Kruskal‒Wallis test was used for variables that were heterogeneous. For data collected longitudinally, mixed-effect analysis of variance was used to analyze the differences between groups, and Greenhouse–Geisser correction was used to adjust sphericity. To correct for multiple testing, the Benjamini–Hochberg method was used to adjust the *P* value. Statistical significance was set at *P* < 0.05.

## RESULTS

### Study population and sample disposition

Overall, we enrolled 121 healthy mother–infant pairs. Since our focus was on evaluating the association of bacterial populations during breastfeeding, we included only pairs where mothers exclusively breastfed and newborns were delivered vaginally. Ultimately, 21 mother–infant pairs met the criteria, and their baseline characteristics are listed in Table 2. On average, the women were 30.52 ± 3.34 years old, weighed 53.41 ± 5.58 kg before pregnancy, and had a gestational age of 39.52 ± 0.98 weeks. Most samples were collected from both mothers and infants at each time point. In total, 54 infant feces and 39 human milk samples were obtained (Table S1). To assess the impact of antibiotic exposure and infant gender, we conducted a stratified analysis comparing the alpha diversity of infant feces between those who received short-term antibiotics at birth or within the first month and those who did not, as well as male and female infants. No significant differences in microbiota profiles were observed between the groups (Tables S2 and S3).

**TABLE 2:** Baseline characteristics of the 21 mother–infant pairs^*a*^

Characteristic	Value
Mothers	
Age at delivery (years)	30.52 ± 3.34
Height (cm)	162.86 ± 5.29
Weight prior to pregnancy (kg)	53.41 ± 5.58
Weight prior to delivery (kg)	68.26 ± 7.91
BMI prior to pregnancy (kg/m^2^)	25.69 ± 2.33
Gestational age (weeks)	39.52 ± 0.98
Parity (*n*)	1.43 ± 0.51
Short-term antibiotics used during/after delivery (*n*)	
Yes	4
No	17
Infants	
Gender (*n*)	
Male	7
Female	14
Birth weight (g)	3,203 ± 732.4
Birth height (cm)	50.21 ± 1.22
Short-term antibiotics used after birth within 1 month	
Yes	4
No	17

^*a*^ The values are the means ± SDs or units of measurement as indicated.

### Dynamic changes in alpha- and beta-diversity in infant feces and human milk

**Fig 1:**
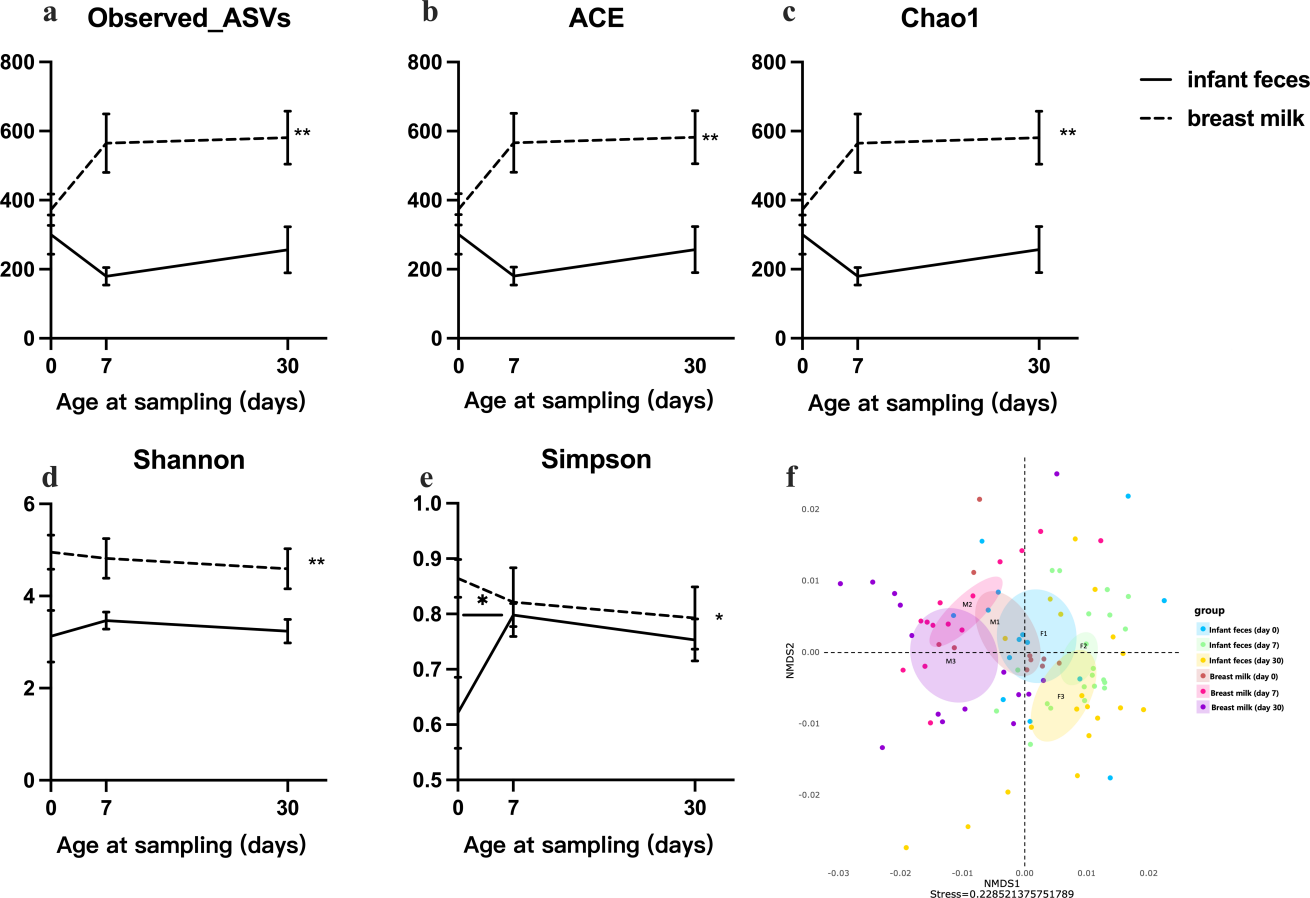
Alpha- and beta-diversity of bacteria in infant feces and human milk during the first month of life. (**a–c**) observed ASV, ACE, and Chao 1 indexes reflecting species richness. (**d and e**) Shannon and Simpson indices reflect species diversity. The Kruskal–Wallis test was used to analyze differences among the four time points in the four groups; the Benjamini-Hochberg method was used to adjust *P* value for multiple testing. Values are presented as mean ± SD; *, adjusted *P* < 0.05‘ **, adjusted *P* < 0.01. (**f**) Beta-diversity plot of infant feces and human milk microbiota at different time points, and two-dimensional NMDS plot based on the Bray-Curtis algorithm and full-level ASVs, where each point represents a sample.

In consideration of sample type and time effects, a mixed-effect analysis of variance was applied to examine the effect of time on alpha diversity indices within each sample type and the effect of sample type across time. The rarefaction curves of alpha-diversity demonstrate that the majority of our samples reached a plateau, suggesting that the sequencing depth was adequate for detecting the majority of microbial diversity in our samples (Fig. S1). As shown in Fig. 1a through e, after adjusting for multiple comparisons, few changes across time were noted, and only the Shannon index of the infant fecal microbiota was higher on day 7 than on day 0 (*P* < 0.05). When diversity across sample types was compared, the overall level of alpha diversity was significantly higher in human milk than in infant feces (all *P* values < 0.05). The observed ASV, ACE, and Chao 1 indices of the human milk samples were greater than those of the infant feces samples at 7 and 30 days (both *P* < 0.05), the Shannon index of the human milk samples was greater than that of the infant feces samples at each follow-up time point (all *P* values < 0.05), and the Simpson index of the human milk samples was greater than that of the infant feces samples on day 0 (*P* < 0.05; Table S4).

Beta-diversity was generated via NMDS analysis on the basis of sample type and time grouping, and the stress of the model was 0.22, which indicated fair goodness of fit. As shown in Fig. 1f, the NMDS plots suggested that the microbiota structure of infant feces and human milk partially overlapped on day 0, and the microbiota structure of the two sample types gradually separated on days 7 and 30 over time. Conversely, the microbiota structure of the same sample type partially overlapped over time.

### Changes in relative microbial abundance in infant feces and breast milk

**Fig 2:**
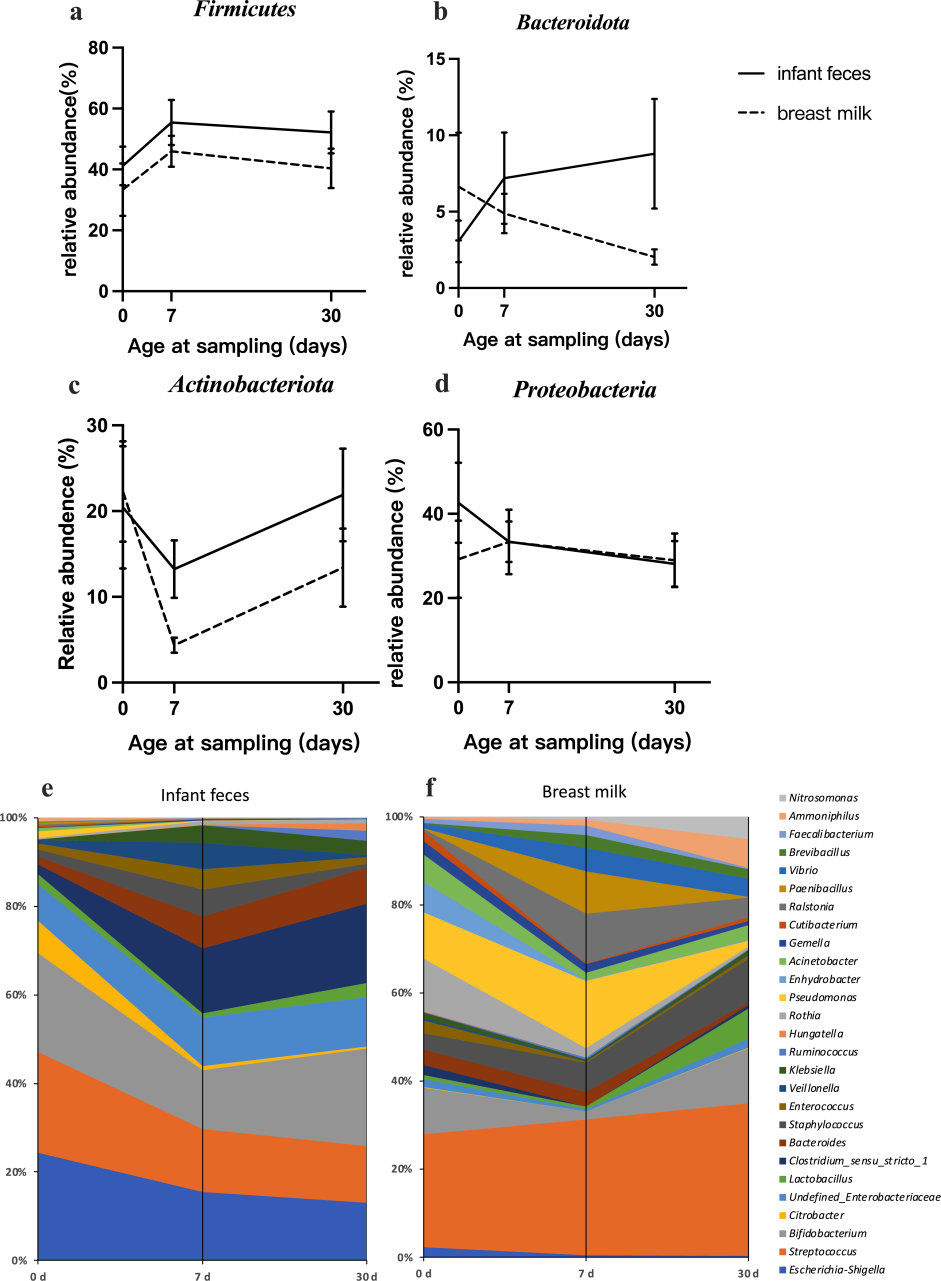
Taxonomic changes at the phylum and genus levels in infant feces and human milk. (a–d) Relative abundance of four dominant phylum in infant feces and breast milk. (e–f) Relative abundance of top ten genus in infant feces and breast milk. Values are presented as mean ± SD.

The taxonomic changes in the top 4 abundant phyla are graphically represented in Fig. 2a through d. Notably, the relative abundance of *Firmicutes* in infant feces and human milk followed a similar trend over time, increasing from day 0 to day 7 and then decreasing by day 30. Furthermore, the relative abundance of *Actinobacteria* in both infant feces and human milk also displayed a consistent pattern, decreasing from day 0 to day 7 and then gradually increasing by day 30.

The relative abundances of the 10 most abundant taxa at the genus level are presented in Fig. 2e and f and in Table S5. Compared with those in infant feces, the most abundant taxa were more stable in human milk, with *Streptococcus* at days 0, 7, and 30 (21.58%, 24.00%, and 25.99%, respectively). However, the highest relative abundance in infant feces at the genus level varied over time, with *Escherichia-Shigella* being the highest on day 0 and day 7 (21.19% and 13.57%, respectively), whereas by day 30, *Bifidobacterium* became the predominant taxon, with a relative abundance of 19.11%. Although both sample types had a unique bacterial community composition, there was a noteworthy similarity. Specifically, the relative abundance of *Bifidobacterium* in infant feces and human milk tended to decrease from day 0 to day 7 but then increased to day 30.

### Potential sources of the bacterial communities and *Bifidobacterium* in infant feces and human milk

**Fig 3:**
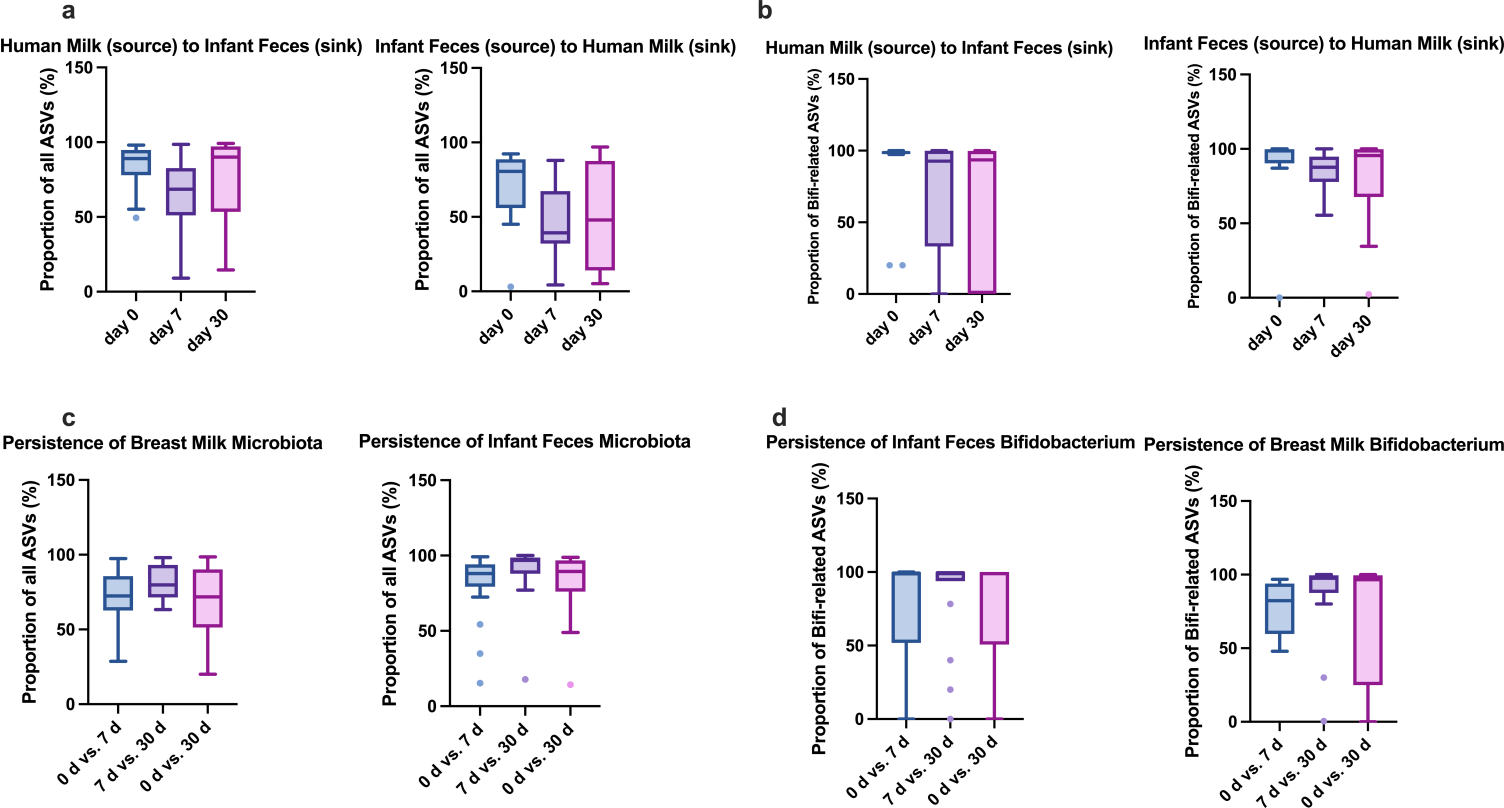
Source of the bacterial community in infant stool and human milk. (**a**) Source tracking analysis based on total ASVs showing shared proportion between infant gut and human milk microbiota. (**b**) Source tracking analysis based on *Bifidobacterium*-related ASVs showing shared proportions between infant gut and human milk microbiota. (**c**) Source tracking analysis based on total ASVs showing persistence in infant gut and human milk microbiota. (**d**) Source tracking analysis based on *Bifidobacterium*-related ASVs showing persistence in infant gut and human milk microbiota. Values are presented as mean ± SD.

Using SourceTracker2 software, we first used total bacterial ASVs to estimate the likely contributions to infant fecal bacterial communities (sink) from human milk (source) and, inversely, the likely contributions to human milk bacterial communities (sink) from infant feces (source). As shown in Fig. 3a, within the first month of life, the estimated overlap of the human milk microbiota with the infant fecal microbiota was consistently higher than that of the infant gut microbiota with human milk. The human milk microbiota was estimated to overlap by mean (SD) 83.71% (15.44%) at birth, decreasing to 63.89% (26.45%) by day 7, and gradually increasing to 77.61% (25.55%) by day 30 with the infant fecal microbiota. Conversely, the overlap of the infant fecal microbiota with human milk microbiota was estimated to be 70.08% (27.78%) at birth, 46.40% (25.64) on day 7, and 50.94% (32.73) on day 30.

Furthermore, to estimate the potential sources of *Bifidobacterium* in infant feces and human milk, Bifidobacterium-related ASVs were utilized, ensuring reliable and consistent signals in the 16S rRNA sequencing data while minimizing classification uncertainty. As shown in Fig. 3b, the proportion of *Bifidobacterium* shared between infant feces and breast milk was consistently higher for infant fecal *Bifidobacterium* compared with that in breast milk. The infant fecal *Bifidobacterium* was estimated to overlap by 87.09% (30.90%), 84.30% (13.91%), and 80.18% (29.60%) with the breast milk microbiota over time. However, the overlap of breast milk *Bifidobacterium* with infant fecal microbiota was 87.58% (28.64%), 67.89% (38.65%), and 60.64% (44.54%) over time.

Additionally, we used SourceTracker2 to assess the persistence of bacterial communities along with *Bifidobacterium* communities within the sample type across three time points (Fig. 3c and d). Overall, the shared proportions value of bacterial communities between day 0 and day 7 were lower than those between days 7 and 30 in both infant feces and human milk (infant feces, mean ± SD: 81.3% ± 21.37% vs. 89.84% ± 18.50%; human milk: 72.22% ± 17.71% vs. 81.05% ± 11.41%). Furthermore, the percentages of *Bifidobacterium* communities shared between day 0 and day 7 were also lower than those between day 7 and day 30 for both infant feces and human milk (infant feces: 75.84% ± 39.97% vs. 85.49% ± 30.30%; human milk: 78.32% ± 17.39% vs. 84.39% ±30.38%).

### Composition of *Bifidobacterium* species isolated from infant feces and human milk

Samples that were complete and adequate from 10 mother–infant pairs were included for isolation, totaling 54 samples (27 infant feces and 27 human milk). The isolation profiles of bifidobacterial strains are shown in Table S6. Overall, the isolation rate of *Bifidobacterium* in infant feces was higher than that in human milk at each time point (infant feces: 11.1%, 44.4%, and 55.6%; human milk: 0, 10%, and 30%), and in both infant feces and human milk samples, the isolation rate gradually increased within the first month of age. A total of 60 bifidobacterial isolates were obtained from 10 vaginally delivered, exclusively breastfed mother–infant pairs. Among them, *B. breve*, *B. longum* subsp. *longum, B. animalis* subsp. *lactis,* and *B. dentium* were obtained from infant feces. *B. breve, B. longum* subsp. *infantis,* and *B. animalis* subsp. *lactis* were obtained from human milk. The concentration of *Bifidobacterium* strains in the infant gut was between 10^6^ CFU/mL and 10^9^ CFU/mL, which is higher than that in human milk, where concentrations ranged from 10^2^ CFU/mL to 10^3^ CFU/mL (Table S7).

Table 3 shows the counts of *Bifidobacterium* isolates in the samples detected from 10 mother–infant pairs. In pair No. 10, *B. breve* was isolated from both infant feces (days 0, 7, and 30) and human milk (day 30). In pair No. 69, *B. longum* subsp. *longum* was isolated from infant feces on days 7 and 30 but was not isolated from human milk. For the remaining eight mother‒infant pairs, *Bifidobacterium* was isolated from either infant feces or human milk at a single time point.

**TABLE 3:** *Bifidobacterium* isolates in the samples detected from 10 mother–infant pairs^*a*^

Species	Pair no.	Sample type	Time postpartum		
			Day 0	Day 7	Day 30
*B. breve*	10	Infant feces	4	9	7
		Human milk	—	—	4
	70	Infant feces	ns	ns	ns
		Human milk	ns	—	6
*B. longum* subsp. *longum*	2-07	Infant feces	—	5	—
		Human milk	ns	—	—
	69	Infant feces	—	8	2
		Human milk	—	—	—
	157	Infant feces	—	3	—
		Human milk	ns	—	—
*B. longum* subsp. *infantis*	153	Infant feces	—	—	—
		Human milk	—	—	1
*B. dentium*	2-18	Infant feces	—	—	2
		Human milk	—	—	—
*B. animalis* subsp*. lactis*	60	Infant feces	—	—	7
		Human milk	—	—	—
	117	Infant feces	—	—	1
		Human milk	—	—	—
	136	Infant feces	—	—	—
		Human milk	—	1	—

^*a*^ "ns" denotes that no sample was collected; "—" denotes that no bifidobacteria were isolated.

### Comparison of MLST profiles of infant-type *Bifidobacterium* isolates from mother–infant pairs

**Fig 4:**
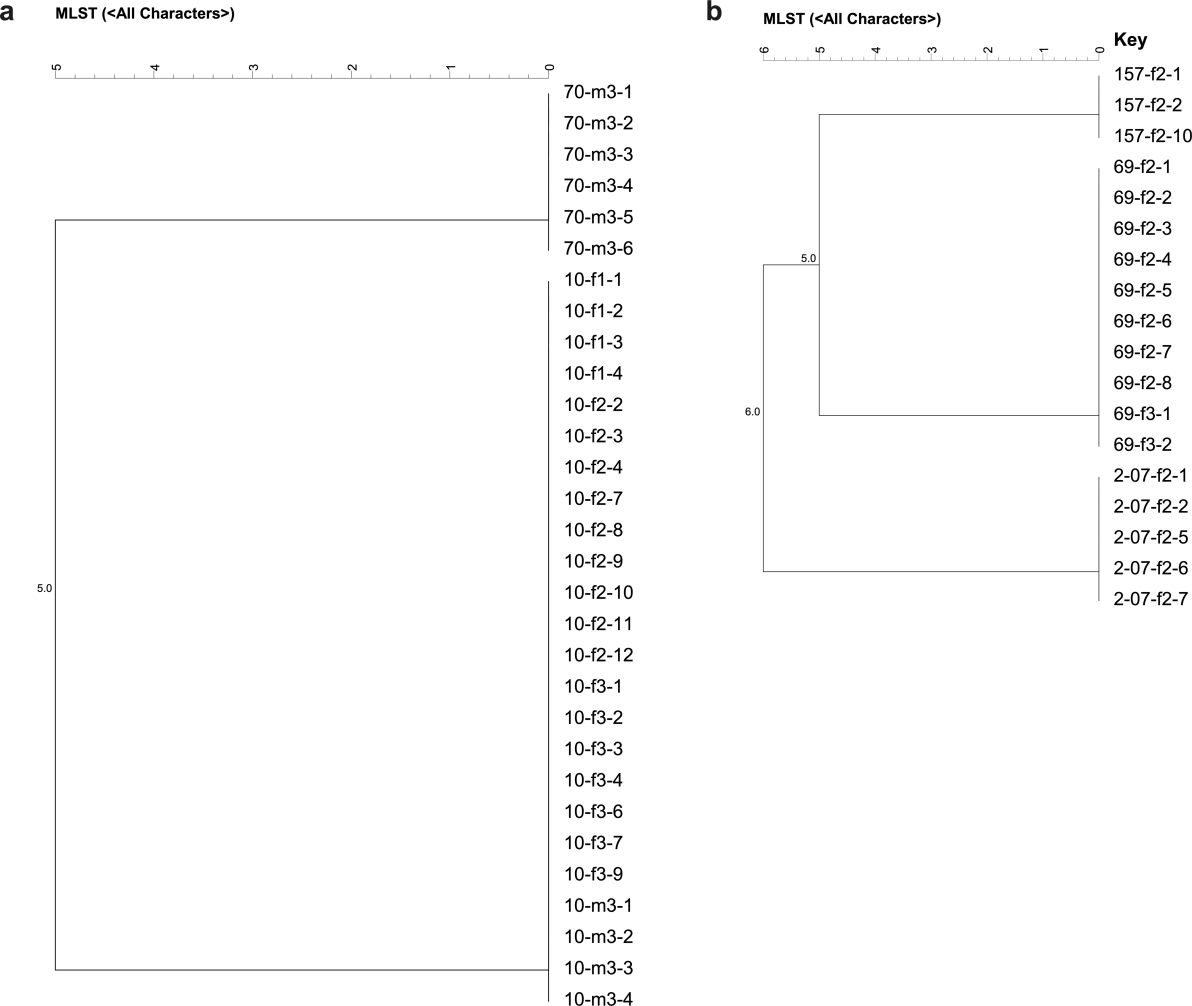
UPGMA dendrogram based on the allelic profiles. (**a**) The genetic relationships between the 2 STs that belong to *B. breve* through MLST typing. (**b**) The genetic relationships between the 3 STs that belong to *B. longum* subsp. *longum* through MLST typing.

The sequences of the seven loci in the 30 *B. breve* isolates and 18 *B. longum* subsp. *longum* isolates were determined. The sequence types (STs) are provided in Table S8. Isolates that had the same STs and were obtained from the same mother–infant pairs were defined as monophyletic strains. Figure 4a shows the dendrogram of *B. breve* isolates. Identical mother–infant monophyletic *B. breve* strains were found in pair No. 10, and the monophyletic strain (ST: BRE-1) was isolated from infant feces on days 0, 7, and 30, and also on day 30 from human milk. Figure 4b shows the dendrogram of *B. longum* subsp. *longum* isolates; identical monophyletic *B. longum* subsp. *longum* strains (ST: LON-2) were isolated from the infant feces of pair No. 69 on days 7 and 30.

## DISCUSSION

Human milk is an early source of bacteria and nutrients introduced to the infant’s gut within a few hours of birth, and the microbial interaction of the two niches consistently occurs. This study relates and compares the differences in the microbiota between infant feces and human milk from vaginally delivered, exclusively breastfed mother–infant pairs, an ideal pattern for the development of infants’ gut microbiota, which can help eliminate the interference of confounding factors. In this study, we observed potential mutual migration between the infant gut microbiota and the human milk microbiota on the basis of alpha diversity. The observed changes in the richness of the infant gut microbiota are consistent with our previous findings (30), indicating that microbial acquisition occurs rapidly within the first month after birth (7). This suggests a dynamic early colonization process, where certain microbes establish and persist in the infant gut. Conversely, the richness of the human milk microbiota increased from day 0 to day 7 and then stabilized. The increased microbial richness may result from breastfeeding. During lactation, the infant’s oral and intestinal microbes migrate to the human milk, thereby increasing the diversity of the human milk microbiota (20, 31). Additionally, the Shannon and Simpson indices reflecting the evenness of microbial communities in infant feces and human milk gradually converged over time, which suggests the reciprocal migration of maternal and infant microbiota during feeding, which is consistent with the findings of a previous study (21). Samples from infant feces and human milk showed distinct clustering across time in beta-diversity, as has been shown previously for other subsets of mother–infant pairs (32, 33). However, our results revealed overlapping clustering on day 0 between the two niches, suggesting that the initial microbes were derived from maternal microbes.

The relative abundances of the top four phyla were *Firmicutes*, *Bacteroidota*, *Actinobacteria,* and *Proteobacteria* in infant feces and human milk. *Firmicutes* were the most predominant phylum in human milk at all three time points, whereas the dominant phylum in infant feces shifted from *Proteobacteria* to *Firmicutes* by 1 month of age. Our results align with those of previous studies on the human milk microbiota, which is generally dominated by *Firmicutes* and *Proteobacteria* (34). Additionally, our previous study found similar trends in infant feces; the relative abundance of *Proteobacteria* in breastfed infants gradually decreased, with *Firmicutes* becoming the most dominant phylum from day 7 and continuing until 3 months of age (30). The variation in *Firmicutes*, *Actinobacteria*, and *Proteobacteria* in infant feces and breast milk showed dynamic changes over time, which may suggest potential microbial migration between the two communities, although further research is needed to definitively establish the directionality of this transfer. Specifically, the relative abundance of *Actinobacteria* in infant feces and human milk decreased from day 0 to day 7, followed by an increase to day 30, and similar trends were found in healthy infants in our previous study (2). The initial decrease and subsequent increase in *Actinobacteriota* during early life might be related to changes in the infant gut environment, showing that the transitions from aerobic to anaerobic in the neonatal gut facilitate the succession of the gut microbiota from facultative anaerobes to obligate anaerobes (35).

Samples from infant feces and human milk presented distinct microbial compositions at the genus level, whereas specific genera exhibited similar dynamic changes. Regardless of geographical differences or analytical methods, current studies generally agree that the most common genera in human milk include *Streptococcus* and *Staphylococcus*, followed by *Bifidobacterium* and *Enterococcus* (36, 37). Our results are similar to those of previous studies; *Streptococcus* was the most abundant genus in human milk at all time points, whereas the relative abundance of *Staphylococcus* increased with increasing lactation stage. The dominant taxa *Streptococcus* and *Staphylococcus* in human milk may be influenced by changes in the mammary environment during the perinatal period. Studies have shown that the rapid proliferation of mammary ducts and alveoli during the perinatal period promotes microbial biofilm formation, and biofilm-related genes have been identified in *Staphylococcus* strains isolated from human milk (38–40). Bacteria are likely in constant exchange between the mother and infant during breastfeeding. Notably, we found that *Bifidobacterium* in infant feces and human milk exhibited the same trend, decreasing from day 0 to day 7 and then increasing. These results are similar to those of previous studies on the dynamics of *Bifidobacterium* in infant feces and human milk (14, 30). The “synchronized” dynamic changes in *Bifidobacterium* in the infant gut and human milk indicate the possibility of mother-to-infant transmission.

Therefore, we next elucidated which direction of microbial contribution is more profound in early infancy, and the proportion and direction of *Bifidobacterium* migration between mothers and infants were investigated in particular. We used SourceTracker2 to analyze the contributions of the microbial communities in both directions. Our results suggest that human milk transfers more bacteria to the infant’s gut within the first month of life, with bacteria from mothers’ milk accounting for 63.89%–77.61% of the gut bacteria in breastfed infants within the first 30 days of life. A previous study reported that 27.7% of infants received bacteria from human milk during the first 30 days of life (9). Korpela and coworkers (41) tracked strain sharing between metagenomes using rare marker single-nucleotide variants (SNVs) and reported that strains from *Actinobacteriota* and *Bacteroidota* are transmitted from the mother and persist for at least 1 year. These early bacterial seeding events may be a mechanism by which breastfeeding protects children. The transmission of *Bifidobacterium* from mothers to their offspring has been considered a pivotal route for *Bifidobacterium* colonization in newborns, although an in-depth evaluation of this process remains limited. To further investigate certain microbial exchanges between the infant gut and human milk, we performed SourceTracker2 analysis using *Bifidobacterium*-related ASVs. Our findings indicate that a substantial proportion of *Bifidobacterium* in human milk overlapped with that from the infant gut, sharing about 80.18% to 84.30% of the total *Bifidobacterium* population. Conversely, *Bifidobacterium* sourced from human milk was detected in 60.64% to 67.89% of infants. This observation does not imply causality but highlights the potential direction of microbial transfer. It is also possible that *Bifidobacterium* thrives in human milk due to its ability to utilize HMOs, which may not be the case for other microbial species.

To further verify the above findings obtained from the cohort study using bioinformatic analysis, we isolated bifidobacteria from samples of human milk and infant feces and performed a genomic comparison to analyze whether the *Bifidobacterium* in the infant intestine was transmitted from the mother’s milk at the strain level. In our present study, the detection rate of *Bifidobacterium* strains was greater in infant feces than in mother’s milk, and both strains tended to increase within the first month. These results concurred with our previous unpublished results in infant fecal *Bifidobacterium* isolates. However, the detection of *Bifidobacterium* strains in human milk has varied across studies. In a study conducted in Inner Mongolia, China, the detection rate of *Bifidobacterium* in colostrum was 4.0% (42). Another study in Japan isolated *Bifidobacterium* from the transitional milk (day 7) and mature milk (day 30) of 12 mothers, and the detection rates were 16.7% and 33.3%, respectively (43). These inconsistent results may be affected by geographical and demographic differences (36, 44).

In our present study, the dominant *Bifidobacterium* isolated from infant feces was *B. breve* and *B. longum* subsp. *longum*, which is similar to the findings of previous studies (45–47). Our study suggests that the dominant colonizers, *B. breve* and *B. longum* subsp. *longum*, in the gut of exclusively breastfed infants may be closely related to the HMOs in human milk. HMOs are the main feeders of infant-type *Bifidobacterium* during the first 6 months in breastfed infants, helping the seeding of *Bifidobacterium* in the infant’s gut and becoming dominant. Infant-type species, including *B. breve*, *B. longum* subsp. *infantis*, *B. longum* subsp. *longum,* and *B. bifidum,* contain specific gene clusters encoding enzymes that are capable of hydrolyzing certain HMOs (48, 49). Although previous studies have suggested that the colonization of *B. longum* subsp. *infantis* is associated with breastfeeding (50), we found no *B. longum* subsp. *infantis* strain present in the infant feces of our cohort; this could either be due to the small sample size or that *B. longum* subsp. *infantis* has gradually diminished in China. Researchers have noted the remarkably low presence of *B. longum* subsp. *infantis* in high-income countries, followed by China and Russia, suggesting that the presence of *Bifidobacterium* is associated with socioeconomic factors (51). Our results generally concurred with previous reports on *Bifidobacterium* species in human milk, which were dominated by *B. breve* (52, 53). The dominant *Bifidobacterium* species in human milk are similar to those in the gut of breastfed infants, suggesting that *Bifidobacterium* may be transmitted between mothers and infants during lactation.

MLST analysis was used to clarify the colonization ability of the specific *Bifidobacterium* strains in the infant’s gut and the direction of their transmission between the mother’s milk and the infant’s intestine. In the present study, the monophyletic strain *B. longum* subsp. *longum* (ST: LON-2) was detected in infant feces on days 7 and 30, and the monophyletic strain *B. breve* (ST: BRE-1) was detected in infant feces within the first month of age at the three following time points. These results indicate that certain infant-type bifidobacterial strains can stably colonize the infant’s gut long-term. Several studies have shown that infant-type *Bifidobacterium* adhere more strongly to infant mucus, which may be associated with the assimilation of mucin glycans and HMOs (16, 54). Furthermore, tight adherence of bifidobacteria may also be related to the conservation of the pilus-encoding locus (55). *B. breve* is known to be one of the predominant species in the infant’s intestinal microbiota and is able to utilize HMOs (56, 57). In our study, monophyletic strains of *B. breve* (ST: BRE-1) were isolated from both infant feces and human milk, and the presence of monophyletic *B. breve* strains occurred earlier in infant feces than in human milk, suggesting that bifidobacterial strains were transmitted from infants to human milk. Our findings align with those of Makino et al. (18)*,* suggesting that only isolates belonging to infant-type bifidobacteria were monophyletic between infant feces and maternal milk and transmitted from the infant’s gut to the mother’s milk during breastfeeding.

However, the origin of *Bifidobacterium* in human milk remains debatable. Two primary pathways have been proposed for the origin of the human milk microbiota: the entero-mammary translocation of maternal gut microbes and the retrograde inoculation from the infant’s oral microbiota. Evidence supporting the entero-mammary route includes the presence of a distinct microbial community in colostrum even before the first infant feeding (58). Meanwhile, the close resemblance between the infant oral microbiota and the human milk microbiota (59, 60) suggests that retrograde transfer during breastfeeding also plays a role. In an aspect of *Bifidobacterium* in human milk, our findings provide intriguing evidence in favor of the retrograde hypothesis, suggesting that *Bifidobacterium* is not the original microbe in human milk but that human milk can transiently harbor specific bifidobacterial strains from infant gut in a retrograde route. Studies have shown that although the oral cavity and gut are directly connected, their microbiomes are distinct. In healthy individuals, the primary oral taxa include *Streptococcus, Veillonella, Gemella, Neisseria, Haemophilus,* and *Rothia* (61). Notably, differences in pH, oxygen levels, nutrient availability, and immune responses create selective pressures, resulting in only a limited number of shared taxa between the two sites (62). However, oxygen-tolerant species exhibit higher transmissibility than strict anaerobes, such as *Streptococcus, Veillonella, Actinomyces,* and *Haemophilus*, and can be transmitted from the oral cavity to the gut, where they establish coherent strain populations along the gastrointestinal tract (63). *Bifidobacterium* is a strictly anaerobic genus, which makes it less likely to be present from oral microbiome or environmental contamination such as skin. Our study also found that the concentration of *Bifidobacterium* in human milk is significantly lower than that in the infant gut. Although the phylogenetic and genomic traits of these strains remain indistinguishable across different human habitats (64), infrared photography has revealed a significant degree of retrograde flow back into the mammary ducts during sucking (65). This evidence provides opportunities for *Bifidobacterium* to migrate from the infant gut to the mother’s milk. However, although the monophyletic *B. breve* strains from the mother–infant pairs in our study did not receive probiotics during the study period, the possibility that the isolated bifidobacterial strains originated from the oral cavity and subsequently colonized both human milk and the infant gut cannot be entirely ruled out. Current studies suggest that the acquisition of oral microbiota is primarily driven by environmental factors (66, 67). Bifidobacterial strains from environmental sources, such as close physical interactions with family members or other infants, may also transiently contribute to the presence of these microbes in the oral cavity (68).

It is important to note that the prevalence of *Bifidobacterium* detected via 16S rRNA sequencing is higher than that observed using culture-based methods. This discrepancy likely stems from technical limitations, as 16S rRNA sequencing cannot differentiate between viable and non-viable bacteria and captures only specific gene fragments rather than full genomes, potentially leading to an overestimation of *Bifidobacterium* abundance in human milk. However, beyond methodological constraints, our findings suggest a biological basis for the presence of *Bifidobacterium* in human milk. Despite differences in detection rates between sequencing and culture-based approaches, the timing of *Bifidobacterium* appearance and its low-level detection in some milk samples through cultivation align with 16S rRNA sequencing results. This supports the notion that *Bifidobacterium* is present in human milk at low levels and may be transmitted retrogradely from the infant gut during breastfeeding rather than being an original resident of human milk.

This study has two main strengths and three limitations. First, comprehensive data were collected from multiple time points of the first month postpartum from the mother–infant cohort, especially the meconium and colostrum, and both sequencing and culture-based methods were used, which provided a detailed understanding of the changes in early-life microbial communities in the infant gut and human milk over time. More importantly, these novel insights into the bidirectional influence between human milk and the infant gut microbiota add significant value and provide new opinions to the literature, as our interesting findings indicate that *Bifidobacterium* may be retrogradely transmitted from the infant gut to the mother’s human milk. Among the key limitations of this study was the limited sample size, as we included only mother‒infant pairs that were vaginally delivered and exclusively breastfed. A larger population is needed in future studies. Owing to the difficulties in sampling, we did not contain maternal vagina, stool, and areolar samples, or infant oral swab samples for sequencing, as these samples may have contributed to additional microbial transmission between mothers and infants other than the human milk we included in this study. Second, shared microbes, particularly *Bifidobacterium*, were found between the mother–infant pairs in human milk and infant feces. The observed overlaps of microbiota profile between human milk and infant feces may not be entirely reflective of true microbial transmission but rather influenced by the resolution and inherent limitations of the sequencing techniques employed. To better understand the specific strains and signaling pathways involved in microbial translocation, animal models should be employed for further investigation. Finally, the detection of *Bifidobacterium* is limited by the inherent constraints of culture-based technologies. These methods may not identify less abundant or fastidious bifidobacterial strains that require specific growth conditions, leading to an incomplete representation of the species diversity in the samples, and 16S rRNA fails in achieving high resolution at the species level. The application of novel culture techniques for culturomics, metagenomics, and ITS sequencing could help detect a broader range of *Bifidobacterium* and its functional profiling.

In conclusion, our microbiome and culture-based data provide valuable insights into the microbial interaction between mothers and their infants, particularly during very early breastfeeding. These data suggest that human milk microbes shared a greater proportion of the overall bacterial population in the infant’s gut. In contrast, the infant gut appears to selectively share a greater proportion of *Bifidobacterium* with human milk. Notably, certain bifidobacterial strains, such as *B. breve*, may be transmitted retrogradely from the infant’s gut to the mother’s human milk. These findings provide valuable insights into the potential dynamics of microbial exchange. The challenge remains in accurately determining the exact source of probiotics isolated from human milk. Further research, including strain-level identification and more rigorous methodologies, is needed to clarify these complex interactions.

## Data Availability

Raw microbiota sequence data have been deposited in the NCBI Sequence Read Archive under accession number PRJNA1273905. The completed STORMS checklist has been made publicly available at Zenodo (https://zenodo.org/records/15629215). Additional data sets generated and analyzed during the current study are also available from the corresponding author upon reasonable request.

## SUPPLEMENTAL MATERIAL

Fig. S1 msystems.00480-25-s0001.tiff Rarefaction analysis using QIIME 2 to assess the alpha diversity across samples at varying sequencing depths.

Supplemental tables. msystems.00480-25-s0002.docx Tables S1 to S8 and legend for Fig. S1.

ASM does not own the copyrights to Supplemental Material that may be linked to, or accessed through, an article. The authors have granted ASM a non-exclusive, world-wide license to publish the Supplemental Material files. Please contact the corresponding author directly for reuse.

## References

[1] Bäckhed F, Roswall J, Peng Y, Feng Q, Jia H, Kovatcheva-Datchary P, Li Y, Xia Y, Xie H, Zhong H, et al.. 2015. Dynamics and stabilization of the human gut microbiome during the first year of life. Cell Host Microbe 17:690–703. doi:10.1016/j.chom.2015.04.004 25974306

[2] Shen X, Wang M, Zhang X, He M, Li M, Cheng G, Wan C, He F. 2019. Dynamic construction of gut microbiota may influence allergic diseases of infants in southwest China. BMC Microbiol 19:123. doi:10.1186/s12866-019-1489-4 31182034 PMC6558729

[3] Wang S, Ryan CA, Boyaval P, Dempsey EM, Ross RP, Stanton C. 2020. Maternal vertical transmission affecting early-life microbiota development. Trends Microbiol 28:28–45. doi:10.1016/j.tim.2019.07.010 31492538

[4] Walker RW, Clemente JC, Peter I, Loos RJF. 2017. The prenatal gut microbiome: are we colonized with bacteria in utero? Pediatr Obes 12 Suppl 1:3–17. doi:10.1111/ijpo.12217 28447406 PMC5583026

[5] Cheng R, Guo J, Zhang Y, Cheng G, Qian W, Wan C, Li M, Marotta F, Shen X, He F. 2021. Impacts of ceftriaxone exposure during pregnancy on maternal gut and placental microbiota and its influence on maternal and offspring immunity in mice. Exp Anim 70:203–217. doi:10.1538/expanim.20-0114 33268669 PMC8150239

[6] Wang S, Zeng S, Egan M, Cherry P, Strain C, Morais E, Boyaval P, Ryan CA, M Dempsey E, Ross RP, Stanton C. 2021. Metagenomic analysis of mother-infant gut microbiome reveals global distinct and shared microbial signatures. Gut Microbes 13:1–24. doi:10.1080/19490976.2021.1911571 PMC8115609 33960282

[7] Ferretti P, Pasolli E, Tett A, Asnicar F, Gorfer V, Fedi S, Armanini F, Truong DT, Manara S, Zolfo M, et al.. 2018. Mother-to-infant microbial transmission from different body sites shapes the developing infant gut microbiome. Cell Host Microbe 24:133–145. doi:10.1016/j.chom.2018.06.005 30001516 PMC6716579

[8] Salminen S, Gibson GR, McCartney AL, Isolauri E. 2004. Influence of mode of delivery on gut microbiota composition in seven year old children. Gut 53:1388–1389. doi:10.1136/gut.2004.041640 PMC1774211 15306608

[9] Pannaraj PS, Li F, Cerini C, Bender JM, Yang S, Rollie A, Adisetiyo H, Zabih S, Lincez PJ, Bittinger K, Bailey A, Bushman FD, Sleasman JW, Aldrovandi GM. 2017. Association between breast milk bacterial communities and establishment and development of the infant gut microbiome. JAMA Pediatr 171:647–654. doi:10.1001/jamapediatrics.2017.0378 28492938 PMC5710346

[10] Bogaert D, van Beveren GJ, de Koff EM, Lusarreta Parga P, Balcazar Lopez CE, Koppensteiner L, Clerc M, Hasrat R, Arp K, Chu MLJN, de Groot PCM, Sanders EAM, van Houten MA, de Steenhuijsen Piters WAA. 2023. Mother-to-infant microbiota transmission and infant microbiota development across multiple body sites. Cell Host Microbe 31:447–460. doi:10.1016/j.chom.2023.01.018 36893737

[11] Wang S, Zeng S, Egan M, Cherry P, Strain C, Morais E, Boyaval P, Ryan CA, Dempsey E, Ross RP, Stanton C. 2021. Metagenomic analysis of mother-infant gut microbiome reveals global distinct and shared microbial signatures. Gut Microbes 13:1911571. doi:10.1080/19490976.2021.1911571 33960282 PMC8115609

[12] Differding MK, Mueller NT. 2020. Human milk bacteria: seeding the infant gut? Cell Host Microbe 28:151–153. doi:10.1016/j.chom.2020.07.017 32791106

[13] Robertson RC, Manges AR, Finlay BB, Prendergast AJ. 2019. The human microbiome and child growth – first 1000 days and beyond. Trends Microbiol 27:131–147. doi:10.1016/j.tim.2018.09.008 30529020

[14] Ding M, Zheng Y, Liu F, Tian F, Ross RP, Stanton C, Yu R, Zhao J, Zhang H, Yang B, Chen W. 2022. Lactation time influences the composition of Bifidobacterium and Lactobacillus at species level in human breast milk. Beneficial Microbes 13:319–330. doi:10.3920/BM2021.0119 35979712

[15] Hidalgo-Cantabrana C, Delgado S, Ruiz L, Ruas-Madiedo P, Sánchez B, Margolles A. 2017. Bifidobacteria and their health-promoting effects. Microbiol Spectr 5. doi:10.1128/microbiolspec.bad-0010-2016 PMC11687494 28643627

[16] Nishiyama K, Yamamoto Y, Sugiyama M, Takaki T, Urashima T, Fukiya S, Yokota A, Okada N, Mukai T. 2017. Bifidobacterium bifidum extracellular sialidase enhances adhesion to the mucosal surface and supports carbohydrate assimilation. mBio 8:e00928-17. doi:10.1128/mBio.00928-17 28974612 PMC5626965

[17] Arboleya S, Ruas-Madiedo P, Margolles A, Solís G, Salminen S, de los Reyes-Gavilán CG, Gueimonde M. 2011. Characterization and in vitro properties of potentially probiotic Bifidobacterium strains isolated from breast-milk. Int J Food Microbiol 149:28–36. doi:10.1016/j.ijfoodmicro.2010.10.036 21109322

[18] Makino H. 2018. Bifidobacterial strains in the intestines of newborns originate from their mothers. Biosci Microbiota Food Health 37:79–85. doi:10.12938/bmfh.18-011 30370191 PMC6200668

[19] Makino H, Kushiro A, Ishikawa E, Kubota H, Gawad A, Sakai T, Oishi K, Martin R, Ben-Amor K, Knol J, Tanaka R. 2013. Mother-to-infant transmission of intestinal bifidobacterial strains has an impact on the early development of vaginally delivered infant’s microbiota. PLoS One 8:e78331. doi:10.1371/journal.pone.0078331 24244304 PMC3828338

[20] Donnet-Hughes A, Perez PF, Doré J, Leclerc M, Levenez F, Benyacoub J, Serrant P, Segura-Roggero I, Schiffrin EJ. 2010. Potential role of the intestinal microbiota of the mother in neonatal immune education. Proc Nutr Soc 69:407–415. doi:10.1017/S0029665110001898 20633308

[21] Gardner H, Kent JC, Hartmann PE, Geddes DT. 2015. Asynchronous milk ejection in human lactating breast. J Hum Lact 31:254–259. doi:10.1177/0890334414568120 25612749

[22] Bolger AM, Lohse M, Usadel B. 2014. Trimmomatic: a flexible trimmer for Illumina sequence data. Bioinformatics 30:2114–2120. doi:10.1093/bioinformatics/btu170 24695404 PMC4103590

[23] Bolyen E, Rideout JR, Dillon MR, Bokulich NA, Abnet CC, Al-Ghalith GA, Alexander H, Alm EJ, Arumugam M, Asnicar F, et al.. 2019. Reproducible, interactive, scalable and extensible microbiome data science using QIIME 2. Nat Biotechnol 37:852–857. doi:10.1038/s41587-019-0209-9 31341288 PMC7015180

[24] Callahan BJ, McMurdie PJ, Rosen MJ, Han AW, Johnson AJA, Holmes SP. 2016. DADA2: high-resolution sample inference from Illumina amplicon data. Nat Methods 13:581–583. doi:10.1038/nmeth.3869 27214047 PMC4927377

[25] Quast C, Pruesse E, Yilmaz P, Gerken J, Schweer T, Yarza P, Peplies J, Glöckner FO. 2013. The SILVA ribosomal RNA gene database project: improved data processing and web-based tools. Nucleic Acids Res 41:D590–D596. doi:10.1093/nar/gks1219 23193283 PMC3531112

[26] Knights D, Kuczynski J, Charlson ES, Zaneveld J, Mozer MC, Collman RG, Bushman FD, Knight R, Kelley ST. 2011. Bayesian community-wide culture-independent microbial source tracking. Nat Methods 8:761–763. doi:10.1038/nmeth.1650 21765408 PMC3791591

[27] Ventura M, Canchaya C, Casale AD, Dellaglio F, Neviani E, Fitzgerald GF, van Sinderen D. 2006. Analysis of bifidobacterial evolution using a multilocus approach. Int J Syst Evol Microbiol 56:2783–2792. doi:10.1099/ijs.0.64233-0 17158978

[28] Santos SR, Ochman H. 2004. Identification and phylogenetic sorting of bacterial lineages with universally conserved genes and proteins. Environ Microbiol 6:754–759. doi:10.1111/j.1462-2920.2004.00617.x 15186354

[29] Delétoile A, Passet V, Aires J, Chambaud I, Butel M-J, Smokvina T, Brisse S. 2010. Species delineation and clonal diversity in four Bifidobacterium species as revealed by multilocus sequencing. Res Microbiol 161:82–90. doi:10.1016/j.resmic.2009.12.006 20060895

[30] Wu S, Ren L, Li J, Shen X, Zhou Q, Miao Z, Jia W, He F, Cheng R. 2022. Breastfeeding might partially contribute to gut microbiota construction and stabilization of propionate metabolism in cesarean-section infants. Eur J Nutr 62:615–631. doi:10.1007/s00394-022-03020-9 36173468

[31] Perez PF, Doré J, Leclerc M, Levenez F, Benyacoub J, Serrant P, Segura-Roggero I, Schiffrin EJ, Donnet-Hughes A. 2007. Bacterial imprinting of the neonatal immune system: lessons from maternal cells? Pediatrics 119:e724–e732. doi:10.1542/peds.2006-1649 17332189

[32] Cabrera-Rubio R, Collado MC, Laitinen K, Salminen S, Isolauri E, Mira A. 2012. The human milk microbiome changes over lactation and is shaped by maternal weight and mode of delivery. Am J Clin Nutr 96:544–551. doi:10.3945/ajcn.112.037382 22836031

[33] Williams JE, Carrothers JM, Lackey KA, Beatty NF, Brooker SL, Peterson HK, Steinkamp KM, York MA, Shafii B, Price WJ, McGuire MA, McGuire MK. 2019. Strong multivariate relations exist among milk, oral, and fecal microbiomes in mother-infant dyads during the first six months postpartum. J Nutr 149:902–914. doi:10.1093/jn/nxy299 31063198 PMC6543206

[34] Togo A, Dufour J-C, Lagier J-C, Dubourg G, Raoult D, Million M. 2019. Repertoire of human breast and milk microbiota: a systematic review. Future Microbiol 14:623–641. doi:10.2217/fmb-2018-0317 31025880

[35] Fanaro S, Chierici R, Guerrini P, Vigi V. 2003. Intestinal microflora in early infancy: composition and development. Acta Paediatr 92:48–55. doi:10.1111/j.1651-2227.2003.tb00646.x 14599042

[36] Hunt KM, Foster JA, Forney LJ, Schütte UME, Beck DL, Abdo Z, Fox LK, Williams JE, McGuire MK, McGuire MA. 2011. Characterization of the diversity and temporal stability of bacterial communities in human milk. PLoS One 6:e21313. doi:10.1371/journal.pone.0021313 21695057 PMC3117882

[37] Fitzstevens JL, Smith KC, Hagadorn JI, Caimano MJ, Matson AP, Brownell EA. 2017. Systematic review of the human milk microbiota. Nut in Clin Prac 32:354–364. doi:10.1177/0884533616670150 27679525

[38] Begović J, Jovčić B, Papić-Obradović M, Veljović K, Lukić J, Kojić M, Topisirović L. 2013. Genotypic diversity and virulent factors of Staphylococcus epidermidis isolated from human breast milk. Microbiol Res 168:77–83. doi:10.1016/j.micres.2012.09.004 23098640

[39] Jiménez E, Delgado S, Maldonado A, Arroyo R, Albújar M, García N, Jariod M, Fernández L, Gómez A, Rodríguez JM. 2008. Staphylococcus epidermidis: a differential trait of the fecal microbiota of breast-fed infants. BMC Microbiol 8:143. doi:10.1186/1471-2180-8-143 18783615 PMC2551609

[40] Delgado S, Arroyo R, Jiménez E, Marín ML, del Campo R, Fernández L, Rodríguez JM. 2009. Staphylococcus epidermidis strains isolated from breast milk of women suffering infectious mastitis: potential virulence traits and resistance to antibiotics. BMC Microbiol 9:82. doi:10.1186/1471-2180-9-82 19422689 PMC2685400

[41] Korpela K, Costea P, Coelho LP, Kandels-Lewis S, Willemsen G, Boomsma DI, Segata N, Bork P. 2018. Selective maternal seeding and environment shape the human gut microbiome. Genome Res 28:561–568. doi:10.1101/gr.233940.117 29496731 PMC5880245

[42] Liu W, Chen M, Duo L, Wang J, Guo S, Sun H, Menghe B, Zhang H. 2020. Characterization of potentially probiotic lactic acid bacteria and bifidobacteria isolated from human colostrum. J Dairy Sci 103:4013–4025. doi:10.3168/jds.2019-17602 32113772

[43] Oki K, Akiyama T, Matsuda K, Gawad A, Makino H, Ishikawa E, Oishi K, Kushiro A, Fujimoto J. 2018. Long-term colonization exceeding six years from early infancy of Bifidobacterium longum subsp. longum in human gut. BMC Microbiol 18:209. doi:10.1186/s12866-018-1358-6 30541439 PMC6292050

[44] Li S-W, Watanabe K, Hsu C-C, Chao S-H, Yang Z-H, Lin Y-J, Chen C-C, Cao Y-M, Huang H-C, Chang C-H, Tsai Y-C. 2017. Bacterial composition and diversity in breast milk samples from mothers living in Taiwan and mainland China. Front Microbiol 8:965. doi:10.3389/fmicb.2017.00965 28611760 PMC5447776

[45] Jost T, Lacroix C, Braegger C, Chassard C. 2013. Assessment of bacterial diversity in breast milk using culture-dependent and culture-independent approaches. Br J Nutr 110:1253–1262. doi:10.1017/S0007114513000597 23507238

[46] Makino H, Kushiro A, Ishikawa E, Muylaert D, Kubota H, Sakai T, Oishi K, Martin R, Ben Amor K, Oozeer R, Knol J, Tanaka R. 2011. Transmission of intestinal Bifidobacterium longum subsp. longum strains from mother to infant, determined by multilocus sequencing typing and amplified fragment length polymorphism. Appl Environ Microbiol 77:6788–6793. doi:10.1128/AEM.05346-11 21821739 PMC3187114

[47] Feehily C, O’Neill IJ, Walsh CJ, Moore RL, Killeen SL, Geraghty AA, Lawton EM, Byrne D, Sanchez-Gallardo R, Nori SRC, Nielsen IB, Wortmann E, Matthews E, O’Flaherty R, Rudd PM, Groeger D, Shanahan F, Saldova R, McAuliffe FM, Van Sinderen D, Cotter PD. 2023. Detailed mapping of Bifidobacterium strain transmission from mother to infant via a dual culture-based and metagenomic approach. Nat Commun 14:3015. doi:10.1038/s41467-023-38694-0 37230981 PMC10213049

[48] James K, O’Connell Motherway M, Penno C, O’Brien RL, van Sinderen D. 2018. Bifidobacterium breve UCC2003 employs multiple transcriptional regulators to control metabolism of particular human milk oligosaccharides. Appl Environ Microbiol 84:e02774-17. doi:10.1128/AEM.02774-17 29500268 PMC5930313

[49] Sakanaka M, Gotoh A, Yoshida K, Odamaki T, Koguchi H, Xiao J-Z, Kitaoka M, Katayama T. 2019. Varied pathways of infant gut-associated Bifidobacterium to assimilate human milk oligosaccharides: prevalence of the gene set and its correlation with bifidobacteria-rich microbiota formation. Nutrients 12:71. doi:10.3390/nu12010071 31888048 PMC7019425

[50] Taft DH, Lewis ZT, Nguyen N, Ho S, Masarweh C, Dunne-Castagna V, Tancredi DJ, Huda MN, Stephensen CB, Hinde K, von Mutius E, Kirjavainen PV, Dalphin J-C, Lauener R, Riedler J, Smilowitz JT, German JB, Morrow AL, Mills DA. 2022. Bifidobacterium species colonization in infancy: a global cross-sectional comparison by population history of breastfeeding. Nutrients 14:1423. doi:10.3390/nu14071423 35406036 PMC9003546

[51] Xu J, Duar RM, Quah B, Gong M, Tin F, Chan P, Sim CK, Tan KH, Chong YS, Gluckman PD, Frese SA, Kyle D, Karnani N. 2024. Delayed colonization of Bifidobacterium spp. and low prevalence of B. infantis among infants of Asian ancestry born in Singapore: insights from the GUSTO cohort study. Front Pediatr 12:1421051. doi:10.3389/fped.2024.1421051 38915873 PMC11194334

[52] Martín R, Jiménez E, Heilig H, Fernández L, Marín ML, Zoetendal EG, Rodríguez JM. 2009. Isolation of Bifidobacteria from breast milk and assessment of the bifidobacterial population by PCR-denaturing gradient gel electrophoresis and quantitative real-time PCR. Appl Environ Microbiol 75:965–969. doi:10.1128/AEM.02063-08 19088308 PMC2643565

[53] Solís G, de los Reyes-Gavilan CG, Fernández N, Margolles A, Gueimonde M. 2010. Establishment and development of lactic acid bacteria and bifidobacteria microbiota in breast-milk and the infant gut. Anaerobe 16:307–310. doi:10.1016/j.anaerobe.2010.02.004 20176122

[54] Harata G, Yoda K, Wang R, Miyazawa K, Sato M, He F, Endo A. 2021. Species- and age/generation-dependent adherence of bifidobacterium bifidum to human intestinal mucus in vitro. Microorganisms 9:542. doi:10.3390/microorganisms9030542 33808003 PMC7998455

[55] O’Connell Motherway M, Zomer A, Leahy SC, Reunanen J, Bottacini F, Claesson MJ, O’Brien F, Flynn K, Casey PG, Moreno Munoz JA, Kearney B, Houston AM, O’Mahony C, Higgins DG, Shanahan F, Palva A, de Vos WM, Fitzgerald GF, Ventura M, O’Toole PW, van Sinderen D. 2011. Functional genome analysis of Bifidobacterium breve UCC2003 reveals type IVb tight adherence (Tad) pili as an essential and conserved host-colonization factor. Proc Natl Acad Sci USA 108:11217–11222. doi:10.1073/pnas.1105380108 21690406 PMC3131351

[56] Mikami K, Takahashi H, Kimura M, Isozaki M, Izuchi K, Shibata R, Sudo N, Matsumoto H, Koga Y. 2009. Influence of maternal bifidobacteria on the establishment of bifidobacteria colonizing the gut in infants. Pediatr Res 65:669–674. doi:10.1203/PDR.0b013e31819ed7a8 19430378

[57] Turroni F, Foroni E, Serafini F, Viappiani A, Montanini B, Bottacini F, Ferrarini A, Bacchini PL, Rota C, Delledonne M, Ottonello S, van Sinderen D, Ventura M. 2011. Ability of Bifidobacterium breve to grow on different types of milk: exploring the metabolism of milk through genome analysis. Appl Environ Microbiol 77:7408–7417. doi:10.1128/AEM.05336-11 21856831 PMC3194849

[58] Damaceno QS, Souza JP, Nicoli JR, Paula RL, Assis GB, Figueiredo HC, Azevedo V, Martins FS. 2017. Evaluation of potential probiotics isolated from human milk and colostrum. Probiotics Antimicro Prot 9:371–379. doi:10.1007/s12602-017-9270-1 28374172

[59] Bisanz JE, Enos MK, PrayGod G, Seney S, Macklaim JM, Chilton S, Willner D, Knight R, Fusch C, Fusch G, Gloor GB, Burton JP, Reid G. 2015. Microbiota at multiple body sites during pregnancy in a rural Tanzanian population and effects of Moringa-supplemented probiotic yogurt. Appl Environ Microbiol 81:4965–4975. doi:10.1128/AEM.00780-15 25979893 PMC4495201

[60] Moossavi S, Sepehri S, Robertson B, Bode L, Goruk S, Field CJ, Lix LM, de Souza RJ, Becker AB, Mandhane PJ, Turvey SE, Subbarao P, Moraes TJ, Lefebvre DL, Sears MR, Khafipour E, Azad MB. 2019. Composition and variation of the human milk microbiota are influenced by maternal and early-life factors. Cell Host Microbe 25:324–335. doi:10.1016/j.chom.2019.01.011 30763539

[61] Kunath BJ, De Rudder C, Laczny CC, Letellier E, Wilmes P. 2024. The oral–gut microbiome axis in health and disease. Nat Rev Microbiol 22:791–805. doi:10.1038/s41579-024-01075-5 39039286

[62] Assimakopoulos SF, Triantos C, Maroulis I, Gogos C. 2018. The role of the gut barrier function in health and disease. Gastroenterol Res 11:261–263. doi:10.14740/gr1053w PMC6089582 30116424

[63] Schmidt TS, Hayward MR, Coelho LP, Li SS, Costea PI, Voigt AY, Wirbel J, Maistrenko OM, Alves RJ, Bergsten E, de Beaufort C, Sobhani I, Heintz-Buschart A, Sunagawa S, Zeller G, Wilmes P, Bork P. 2019. Extensive transmission of microbes along the gastrointestinal tract. Elife 8:e42693. doi:10.7554/eLife.42693 30747106 PMC6424576

[64] Wong CB, Odamaki T, Xiao J. 2020. Insights into the reason of Human-Residential Bifidobacteria (HRB) being the natural inhabitants of the human gut and their potential health-promoting benefits. FEMS Microbiol Rev 44:369–385. doi:10.1093/femsre/fuaa010 32319522 PMC7326374

[65] Xu R, McLoughlin G, Nicol M, Geddes D, Stinson L. 2024. Residents or tourists: is the lactating mammary gland colonized by residential microbiota? Microorganisms 12:1009. doi:10.3390/microorganisms12051009 38792838 PMC11123721

[66] Mukherjee C, Moyer CO, Steinkamp HM, Hashmi SB, Beall CJ, Guo X, Ni A, Leys EJ, Griffen AL. 2021. Acquisition of oral microbiota is driven by environment, not host genetics. Microbiome 9:54. doi:10.1186/s40168-020-00986-8 33622378 PMC7903647

[67] Shaw L, Ribeiro ALR, Levine AP, Pontikos N, Balloux F, Segal AW, Roberts AP, Smith AM. 2017. The human salivary microbiome is shaped by shared environment rather than genetics: evidence from a large family of closely related individuals. mBio 8:e01237-17. doi:10.1128/mBio.01237-17 28900019 PMC5596345

[68] Valles-Colomer M, Blanco-Míguez A, Manghi P, Asnicar F, Dubois L, Golzato D, Armanini F, Cumbo F, Huang KD, Manara S, et al.. 2023. The person-to-person transmission landscape of the gut and oral microbiomes. Nature 614:125–135. doi:10.1038/s41586-022-05620-1 36653448 PMC9892008

