# Supplementary figures and images for: Early life bifidobacterial mother–infant transmission: greater contribution from the infant gut to human milk revealed by microbiomic and culture-based methods
Source: mSystems. 2025 Jun 25;10(7):e00480-25. doi: 10.1128/msystems.00480-25 (PMC12282193; doi:10.1128/msystems.00480-25)

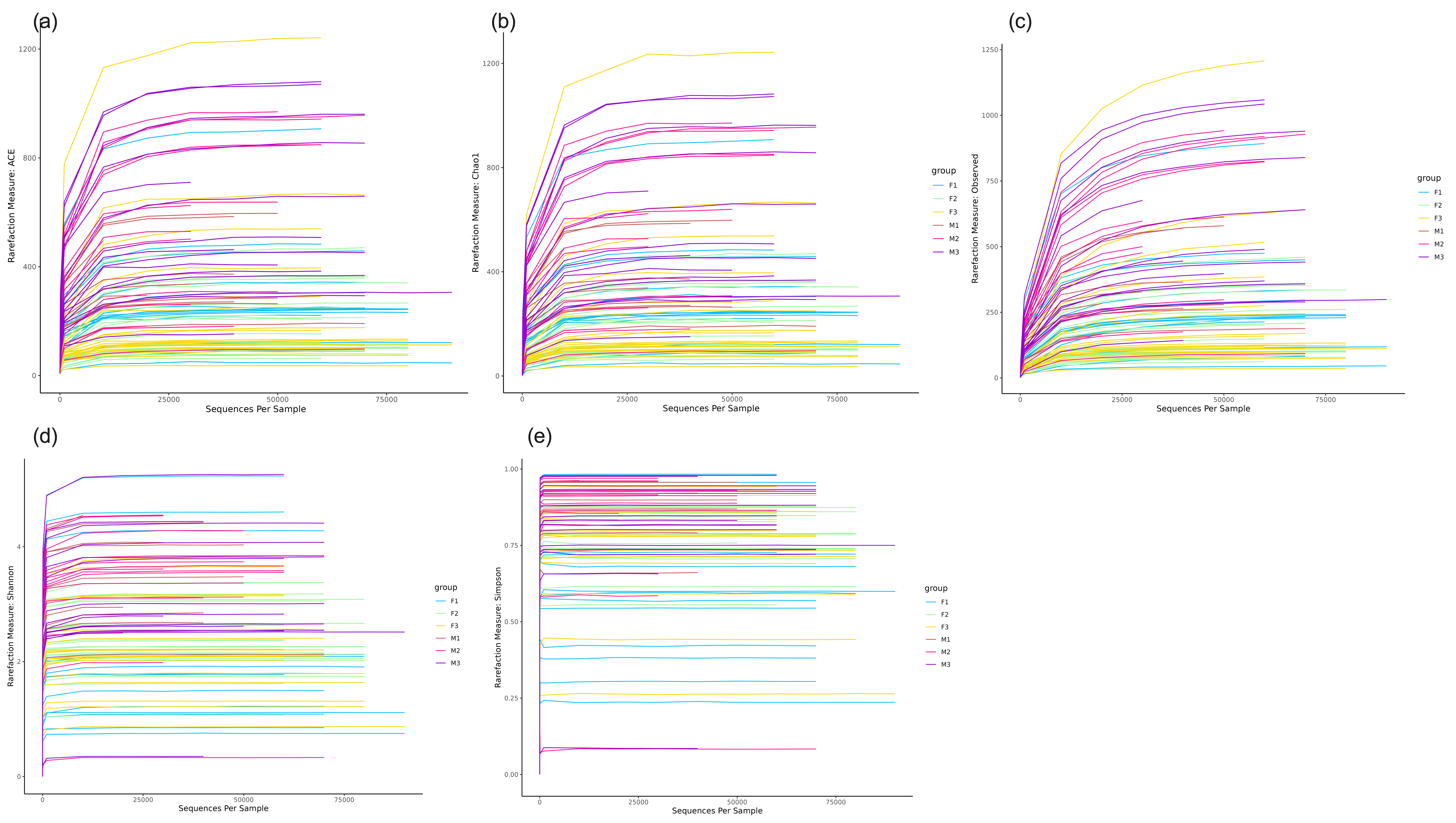

Supplement: Fig. S1 — Rarefaction analysis using QIIME 2 to assess the alpha diversity across samples at varying sequencing depths. [file msystems.00480-25-s0001.tiff]
