# Supplementary material for: Early life bifidobacterial mother–infant transmission: greater contribution from the infant gut to human milk revealed by microbiomic and culture-based methods
Source: mSystems. 2025 Jun 25;10(7):e00480-25. doi: 10.1128/msystems.00480-25 (PMC12282193; doi:10.1128/msystems.00480-25)
Supplement: Supplemental tables. — Tables S1 to S8 and legend for Fig. S1. [file msystems.00480-25-s0002.docx]

**Supplementary Table S1 Samples of infant feces and human milk at each time point (n)**

|  | Day 0 | Day 7 | Day 30 | Total |
| --- | --- | --- | --- | --- |
| Infant feces | 14 | 21 | 19 | 54 |
| Human milk | 10 | 14 | 15 | 39 |
| Total | 24 | 35 | 34 | 93 |

**Supplementary Table S2 Comparison of alpha diversity in antibiotic use and non-antibiotic use participants**

| Sample type | Time | Alpha diversity | Antibiotic use | | *P* value |
| --- | --- | --- | --- | --- | --- |
|  |  |  | Use | Non-use |  |
| Infant feces | Day 0 | ACE | 392.10±454.24 | 276.05±122.09 | 0.659 |
|  |  | Chao 1 | 391.69±453.96 | 275.25±121.57 | 0.659 |
|  |  | Observed | 391.67±453.94 | 275.09±121.35 | 0.659 |
|  |  | Shannon | 3.91±3.20 | 2.92±1.86 | 0.769 |
|  |  | Simpson | 0.77±0.20 | 0.58±0.24 | 0.126 |
|  | Day 7 | ACE | 169.57±128.47 | 182.95±119.75 | 1.000 |
|  |  | Chao 1 | 169.10±128.13 | 182.37±119.19 | 0.965 |
|  |  | Observed | 169.00±127.94 | 182.24±118.98 | 0.965 |
|  |  | Shannon | 3.30±1.40 | 3.51±0.72 | 0.829 |
|  |  | Simpson | 0.77±0.16 | 0.81±0.08 | 0.965 |
|  | Day 30 | ACE | 98.29±56.21 | 299.46±315.38 | 0.080 |
|  |  | Chao 1 | 98.01±55.96 | 299.02±315.51 | 0.080 |
|  |  | Observed | 98.00±55.96 | 298.93±315.54 | 0.080 |
|  |  | Shannon | 2.59±0.58 | 3.41±1.18 | 0.152 |
|  |  | Simpson | 0.72±0.09 | 0.76±0.18 | 0.262 |
| Human milk | Day 0 | ACE | 288.45±132.41 | 394.87±145.14 | 0.533 |
|  |  | Chao 1 | 286.84±131.92 | 394.17±145.22 | 0.533 |
|  |  | Observed | 286.00±131.52 | 394.00±145.32 | 0.533 |
|  |  | Shannon | 4.72±2.33 | 5.01±0.99 | 0.889 |
|  |  | Simpson | 0.86±0.17 | 0.87±0.10 | 1.000 |
|  | Day 7 | ACE | 849.60±181.34 | 489.18±309.37 | 0.088 |
|  |  | Chao 1 | 848.10±181.16 | 488.19±308.43 | 0.088 |
|  |  | Observed | 838.00±181.12 | 488.09±308.30 | 0.088 |
|  |  | Shannon | 5.42±0.13 | 4.65±1.80 | 0.291 |
|  |  | Simpson | 0.90±0.02 | 0.80±0.26 | 1.000 |
|  | Day 30 | ACE | 582.18±284.94 | 546.89±273.96 | 0.267 |
|  |  | Chao 1 | 580.96±284.57 | 545.67±273.66 | 0.267 |
|  |  | Observed | 580.80±284.53 | 545.50±273.67 | 0.267 |
|  |  | Shannon | 4.59±1.58 | 4.53±1.73 | 0.800 |
|  |  | Simpson | 0.79±0.19 | 0.79±0.23 | 0.933 |

Independent-Samples Mann-Whitney U Test was used for comparison of alpha diversity in antibiotic use and non-antibiotic use participants

**Supplementary Table S3 Comparison of alpha diversity between male and female infants**

| Time | Alpha diversity | Gender | | *P* value |
| --- | --- | --- | --- | --- |
|  |  | Male | Female |  |
| Day 0 | ACE | 271.84±52.34 | 317.07±268.31 | 0.636 |
|  | Chao 1 | 271.25±52.33 | 316.28±267.96 | 0.637 |
|  | Observed | 271.20±52.33 | 316.11±267.86 | 0.638 |
|  | Shannon | 2.31±0.62 | 3.58±2.52 | 0.183 |
|  | Simpson | 0.57±0.16 | 0.65±0.28 | 0.476 |
| Day 7 | ACE | 139.33±103.88 | 200.94±123.13 | 0.249 |
|  | Chao 1 | 138.92±103.79 | 200.31±122.45 | 0.249 |
|  | Observed | 139.86±103.80 | 200.14±122.16 | 0.250 |
|  | Shannon | 3.10±0.99 | 3.65±0.74 | 0.224 |
|  | Simpson | 0.77±0.12 | 0.81±0.08 | 0.453 |
| Day 30 | ACE | 237.26±216.31 | 266.27±328.17 | 0.822 |
|  | Chao 1 | 236.91±216.47 | 265.84±328.24 | 0.823 |
|  | Observed | 236.83±216.52 | 265.77±328.24 | 0.823 |
|  | Shannon | 2.73±0.73 | 3.47±1.21 | 0.125 |
|  | Simpson | 0.69±0.22 | 0.78±0.14 | 0.404 |

Independent-Samples Mann-Whitney U Test was used for comparison of alpha diversity in antibiotic use and non-antibiotic use participants

**Supplementary Table S4 Alpha diversity of infant feces and human milk bacteria at each time point**

| Alpha diversity | Time postpartum | | |
| --- | --- | --- | --- |
|  | Day 0 | Day 7 | Day 30 |
| Observed_ASVs |  |  |  |
| Infant feces | 300.07±57.00 | 179.71±25.64 | 256.63±66.90 |
| Human milk | 372.40±45.19 | 565.21±85.24^*^ | 580.80±76.69^*^ |
| ACE |  |  |  |
| Infant feces | 300.92±57.10 | 180.40±25.80 | 257.11±66.88 |
| Human milk | 373.59±45.11 | 566.41±85.45^*^ | 582.18±76.76^*^ |
| Chao1 |  |  |  |
| Infant feces | 300.20±57.03 | 179.84±25.69 | 256.70±66.90 |
| Human milk | 372.70±45.15 | 565.31±85.24^*^ | 580.93±76.69^*^ |
| Shannon |  |  |  |
| Infant feces | 3.13±0.56 | 3.47±0.19 | 3.24±0.26 |
| Human milk | 4.95±0.37^*^ | 4.82±0.43 | 4.59±0.44 |
| Simpson |  |  |  |
| Infant feces | 0.62±0.06 | 0.80±0.02 | 0.75±0.04 |
| Human milk | 0.86±0.03^*^ | 0.82±0.06 | 0.79±0.06 |

The Kruskal–Wallis test was used to analyze differences among the four time points in the four groups, the Benjamini-Hochberg method was used to adjust *P* value for multiple testing. Values are presented as mean ± SD, * adjusted *P* < 0.05

**Supplementary Table S5 The average relative abundance (%) of top 10 taxa at genus level in infant feces and human milk at 1 month of age**

| Sample type | Day 0 | | Day 7 | | Day 30 | |
| --- | --- | --- | --- | --- | --- | --- |
|  | genus | % | genus | % | genus | % |
| Infant feces | *Escherichia-Shigella* | 21.19 | *Escherichia-Shigella* | 13.57 | *Bifidobacterium* | 19.11 |
|  | *Streptococcus* | 19.71 | *Clostridium_sensu_stricto_1* | 12.82 | *Clostridium_sensu_stricto_1* | 15.54 |
|  | *Bifidobacterium* | 19.38 | *Streptococcus* | 12.28 | *Escherichia-Shigella* | 11.40 |
|  | *Undefined_Enterobacteriaceae* | 7.06 | *Bifidobacterium* | 11.66 | *Streptococcus* | 11.17 |
|  | *Citrobacter* | 6.44 | *Undefined_Enterobacteriaceae* | 9.60 | *Undefined_Enterobacteriaceae* | 9.67 |
|  | *Lactobacillus* | 2.01 | *Bacteroides* | 6.23 | *Bacteroides* | 7.09 |
|  | *Clostridium_sensu_stricto_1* | 1.82 | *Staphylococcus* | 5.36 | *Klebsiella* | 2.87 |
|  | *Bacteroides* | 1.60 | *Veillonella* | 5.17 | *Lactobacillus* | 2.77 |
|  | *Staphylococcus* | 1.40 | *Enterococcus* | 3.93 | *Ruminococcus* | 1.93 |
|  | *Pseudomonas* | 1.23 | *Klebsiella* | 3.52 | *Hungatella* | 1.47 |
| Human milk | *Streptococcus* | 21.58 | *Streptococcus* | 24.00 | *Streptococcus* | 25.99 |
|  | *Rothia* | 10.07 | *Pseudomonas* | 11.95 | *Bifidobacterium* | 9.44 |
|  | *Bifidobacterium* | 8.94 | *Ralstonia* | 8.85 | *Staphylococcus* | 7.42 |
|  | *Pseudomonas* | 8.83 | *Paenibacillus* | 7.43 | *Lactobacillus* | 5.16 |
|  | *Enhydrobacter* | 5.83 | *Staphylococcus* | 5.11 | *Ammoniphilus* | 4.85 |
|  | *Acinetobacter* | 5.28 | *Vibrio* | 4.20 | *Nitrosomonas* | 3.83 |
|  | *Staphylococcus* | 3.06 | *Bacteroides* | 2.62 | *Ralstonia* | 3.32 |
|  | *Bacteroides* | 3.06 | *Brevibacillus* | 2.30 | *Vibrio* | 3.06 |
|  | *Gemella* | 2.57 | *Rothia* | 1.61 | *Acinetobacter* | 2.60 |
|  | *Enterococcus* | 2.46 | *Faecalibacterium* | 1.61 | *Brevibacillus* | 1.67 |

**Supplementary Table S6 Isolation profile of *Bifidobacterium* in samples from 10 mother–infant pairs**

| Time (d) | Infant feces | | | Human milk | | |
| --- | --- | --- | --- | --- | --- | --- |
|  | Sample (n) | Positive sample (n) | Detection rate (%) | Sample (n) | Positive sample (n) | Detection rate (%) |
| 0 | 9 | 1 | 11.1 | 7 | 0 | 0 |
| 7 | 9 | 4 | 44.4 | 10 | 1 | 10 |
| 30 | 9 | 5 | 55.6 | 10 | 3 | 30 |
| Total | 27 | 10 | 37 | 27 | 4 | 14.8 |

**Supplementary Table S7 Distribution of 60 strains of *Bifidobacterium* species**

| Sample Source | Species | Counts (%) | Concentration (log_10_ CFU/ ml) | | |
| --- | --- | --- | --- | --- | --- |
|  |  |  | Day 0 | Day 7 | Day 30 |
| Infant feces | *B. breve* | 27 (49.09) | 9 | 9 | 9 |
|  | *B. longum* subsp. *longum* | 18 (32.73) | 0 | 7.3 | 6 |
|  | *B. dentium* | 2 (3.64) | 0 | 0 | 6 |
|  | *B. animalis* subsp. *lactis* | 8 (14.55) | 0 | 0 | 6.5 |
| Human milk | *B. breve* | 10 (83.33) | 0 | 0 | 2 |
|  | *B. animalis* subsp. *lactis* | 1 (8.33) | 0 | 0 | 3 |
|  | *B. longum* subsp. *infantis* | 1 (8.33) | 0 | 2 | 0 |

**Supplementary Table S8 The sequence types (STs) of *B.breve* and *B. longum* subsp. *longum***

| Key | MLST ST | *ileS* | *clpC* | *fusA* | *gyrB* | *purF* | *rplB* | *rpoB* |
| --- | --- | --- | --- | --- | --- | --- | --- | --- |
| 10-f1-1 | BRE-1 | 1 | 1 | 1 | 1 | 1 | 1 | 1 |
| 10-f1-2 | BRE-1 | 1 | 1 | 1 | 1 | 1 | 1 | 1 |
| 10-f1-3 | BRE-1 | 1 | 1 | 1 | 1 | 1 | 1 | 1 |
| 10-f1-4 | BRE-1 | 1 | 1 | 1 | 1 | 1 | 1 | 1 |
| 10-f2-2 | BRE-1 | 1 | 1 | 1 | 1 | 1 | 1 | 1 |
| 10-f2-3 | BRE-1 | 1 | 1 | 1 | 1 | 1 | 1 | 1 |
| 10-f2-4 | BRE-1 | 1 | 1 | 1 | 1 | 1 | 1 | 1 |
| 10-f2-7 | BRE-1 | 1 | 1 | 1 | 1 | 1 | 1 | 1 |
| 10-f2-8 | BRE-1 | 1 | 1 | 1 | 1 | 1 | 1 | 1 |
| 10-f2-9 | BRE-1 | 1 | 1 | 1 | 1 | 1 | 1 | 1 |
| 10-f2-10 | BRE-1 | 1 | 1 | 1 | 1 | 1 | 1 | 1 |
| 10-f2-11 | BRE-1 | 1 | 1 | 1 | 1 | 1 | 1 | 1 |
| 10-f2-12 | BRE-1 | 1 | 1 | 1 | 1 | 1 | 1 | 1 |
| 10-f3-1 | BRE-1 | 1 | 1 | 1 | 1 | 1 | 1 | 1 |
| 10-f3-2 | BRE-1 | 1 | 1 | 1 | 1 | 1 | 1 | 1 |
| 10-f3-3 | BRE-1 | 1 | 1 | 1 | 1 | 1 | 1 | 1 |
| 10-f3-4 | BRE-1 | 1 | 1 | 1 | 1 | 1 | 1 | 1 |
| 10-f3-6 | BRE-1 | 1 | 1 | 1 | 1 | 1 | 1 | 1 |
| 10-f3-7 | BRE-1 | 1 | 1 | 1 | 1 | 1 | 1 | 1 |
| 10-f3-9 | BRE-1 | 1 | 1 | 1 | 1 | 1 | 1 | 1 |
| 10-m3-1 | BRE-1 | 1 | 1 | 1 | 1 | 1 | 1 | 1 |
| 10-m3-2 | BRE-1 | 1 | 1 | 1 | 1 | 1 | 1 | 1 |
| 10-m3-3 | BRE-1 | 1 | 1 | 1 | 1 | 1 | 1 | 1 |
| 10-m3-4 | BRE-1 | 1 | 1 | 1 | 1 | 1 | 1 | 1 |
| 70-m3-1 | BRE-2 | 1 | 2 | 2 | 2 | 2 | 1 | 4 |
| 70-m3-2 | BRE-2 | 1 | 2 | 2 | 2 | 2 | 1 | 4 |
| 70-m3-3 | BRE-2 | 1 | 2 | 2 | 2 | 2 | 1 | 4 |
| 70-m3-4 | BRE-2 | 1 | 2 | 2 | 2 | 2 | 1 | 4 |
| 70-m3-5 | BRE-2 | 1 | 2 | 2 | 2 | 2 | 1 | 4 |
| 70-m3-6 | BRE-2 | 1 | 2 | 2 | 2 | 2 | 1 | 4 |
| 2-07-f2-1 | LON-1 | 2 | 3 | 3 | 3 | 3 | 2 | 3 |
| 2-07-f2-2 | LON-1 | 2 | 3 | 3 | 3 | 3 | 2 | 3 |
| 2-07-f2-5 | LON-1 | 2 | 3 | 3 | 3 | 3 | 2 | 3 |
| 2-07-f2-6 | LON-1 | 2 | 3 | 3 | 3 | 3 | 2 | 3 |
| 2-07-f2-7 | LON-1 | 2 | 3 | 3 | 3 | 3 | 2 | 3 |
| 69-f2-1 | LON-2 | 3 | 4 | 4 | 4 | 4 | 2 | 2 |
| 69-f2-2 | LON-2 | 3 | 4 | 4 | 4 | 4 | 2 | 2 |
| 69-f2-3 | LON-2 | 3 | 4 | 4 | 4 | 4 | 2 | 2 |
| 69-f2-4 | LON-2 | 3 | 4 | 4 | 4 | 4 | 2 | 2 |
| 69-f2-5 | LON-2 | 3 | 4 | 4 | 4 | 4 | 2 | 2 |
| 69-f2-6 | LON-2 | 3 | 4 | 4 | 4 | 4 | 2 | 2 |
| 69-f2-7 | LON-2 | 3 | 4 | 4 | 4 | 4 | 2 | 2 |
| 69-f2-8 | LON-2 | 3 | 4 | 4 | 4 | 4 | 2 | 2 |
| 69-f3-1 | LON-2 | 3 | 4 | 4 | 4 | 4 | 2 | 2 |
| 69-f3-2 | LON-2 | 3 | 4 | 4 | 4 | 4 | 2 | 2 |
| 157-f2-1 | LON-3 | 4 | 5 | 5 | 5 | 3 | 2 | 2 |
| 157-f2-2 | LON-3 | 4 | 5 | 5 | 5 | 3 | 2 | 2 |
| 157-f2-10 | LON-3 | 4 | 5 | 5 | 5 | 3 | 2 | 2 |

**Supplementary Figure S1.**

**Rarefaction curves for all samples.** Rarefaction analysis was performed using QIIME 2 to assess the alpha diversity across samples at varying sequencing depths.
